# Mechanical stimulation of the foot sole in a supine position for ground reaction force simulation

**DOI:** 10.1186/1743-0003-11-159

**Published:** 2014-11-28

**Authors:** Juan Fang, Aleksandra Vuckovic, Sujay Galen, Bernard A Conway, Kenneth J Hunt

**Affiliations:** Institute for Rehabilitation and Performance Technology, Division of Mechanical Engineering, Department of Engineering and Information Technology, Bern University of Applied Sciences, Burgdorf, 3400 Switzerland; Centre for Rehabilitation Engineering, School of Engineering, University of Glasgow, Glasgow, UK; Biomedical Engineering, University of Strathclyde, Glasgow, UK; Physical Therapy Program, Wayne State University, Detroit, USA

**Keywords:** Foot sole stimulation, Dynamic shoe platform, Reflex, Ground reaction force simulation

## Abstract

**Background:**

To promote early rehabilitation of walking, gait training can start even when patients are on bed rest. Supine stepping in the early phase after injury is proposed to maximise the beneficial effects of gait restoration. In this training paradigm, mechanical loading on the sole of the foot is required to mimic the ground reaction forces that occur during overground walking. A pneumatic shoe platform was developed to produce adjustable forces on the heel and the forefoot with an adaptable timing. This study aimed to investigate the stimulation parameters of the shoe platform to generate walking-like loading on the foot sole, while avoiding strong reflexes.

**Methods:**

This study evaluated this platform in ten able-bodied subjects in a supine position. The platform firstly produced single-pulse stimulation on the heel or on the forefoot to determine suitable stimulation parameters, then it produced cyclic stimulation on the heel and the forefoot to simulate the ground reaction forces that occur at different walking speeds. The ankle angle and electromyography (EMG) in the tibialis anterior (TA) and soleus (SOL) muscles were recorded. User feedback was collected.

**Results:**

When the forefoot or/and the heel were stimulated, reflexes were observed in the lower leg muscles, and the amplitude increased with force. Single-pulse stimulation showed that a fast-rising force significantly increased the reflex amplitudes, with the possibility of inducing ankle perturbation. Therefore a slow-rising force pattern was adopted during cyclic stimulation for walking. The supine subjects perceived loading sensation on the foot sole which was felt to be similar to the ground reaction forces during upright walking. The EMG generally increased with force amplitude, but no reflex-induced ankle perturbations were observed. The mean change in the ankle joint induced by the stimulation was about 1°.

**Conclusions:**

The rate of force increase should be carefully adjusted for simulation of walking-like loading on the foot sole. It is concluded that the dynamic shoe platform provides adjustable mechanical stimulation on the heel and the forefoot in a supine position and has technical potential for simulation of ground reaction forces that occur during walking.

**Electronic supplementary material:**

The online version of this article (doi:10.1186/1743-0003-11-159) contains supplementary material, which is available to authorized users.

## Background

Patients with injury to or disease of the central nervous system often have impaired lower limb function and require bed rest in the acute phase of recovery. In order to provide gait training at this stage, a gait orthosis for early rehabilitation of walking was proposed for stepping in a supine position [1]. This has a linkage system to generate walking-like motion in the lower limbs of a supine subject [1, 2]. Effective gait training requires integration of proprioceptive sensory input from the joints of the lower extremity and load interactions between the foot and the ground [3]. Apart from the coordinated leg movement produced by the gait orthosis [1], suitable stimulation of the load receptors in the lower limbs is another key requirement of such a gait orthosis for successful neurological recovery [4].

The sensory loading input from the foot sole has an important role in modulating walking patterns and is beneficial for relearning of walking. During overground walking, load receptors in the foot sole detect changes of the body’s centre of mass (somatosensory input), which provides proprioceptive feedback for maintenance of balance [5]. The cutaneous mechanoreceptors on the foot sole further detect ground surfaces [3], and offer information for modulation of walking patterns [6]. In contrast, when the feet are unloaded, the neural transmission for gait control is disrupted. Air stepping without ground forces on the foot produces walking with variant kinematics [7]. After spinal cord injury, patients produce increased muscle activity if the limb loading increases during walking training [8]. In order to practise stepping in a supine position in the gait orthosis described in [1], an appropriate loading input should thus be implemented to mimic the ground reaction forces that occur during overground walking [4].

There are several types of device for foot-pressure stimulation, but further investigation is required on the target stimulation pattern and intensity. Vibrating insoles were proposed to stimulate the foot sole for somatosensory feedback via vibrating tactile actuators [9, 10]. Air-inflated boots were designed to apply pressure on the foot sole with increased neuromuscular activation in the lower limbs [11]. However, these devices were not specifically designed to simulate the ground reaction force patterns. The force was applied simultaneously on the whole foot sole, which is different from the adaptable force pattern during overground walking. To simulate walking-like loading, pneumatic rubber insoles [12] and stimulative shoes [13] were designed. The pneumatic rubber insoles include two rubber chambers, which take about 0.2 s to produce a target pressure on the foot sole [12]. The stimulative shoe uses a series of cylinder-actuated rods, which allows fast stimulation on the foot sole in less than 0.1 s [13]. During stimulation on the fool sole, too-low intensity, such as slow pressing through rubber chambers, might produce limited haptic sensation, while too strong and fast stimulation might induce reflexes and even reflex-induced movement of the lower limb [14, 15]. The haptic sensation was documented through user feedback [12], but the EMG response directly induced by these devices was not reported.

In order to mimic ground reaction forces for users practising gait in a supine position, a dynamic shoe platform was designed in the present work using pneumatic cylinders. It was expected that the mechanical stimulation of the foot soles would produce reflexes, in addition to haptic sensation [14–16]. Strong reflexes might induce ankle movement, resulting in the ankle trajectory deviating from the kinematics that the gait orthosis is programmed to simulate. To facilitate training and to prevent injuries to potential users, the gait orthosis needs to be able to dampen or restrict these strong reflex responses by modulating the force patterns applied to the sole of the foot.

The aim of this work was to investigate the stimulation parameters of the shoe platform for walking-like loading simulation among able-bodied subjects in a supine position. Lower leg muscle activity (EMG) and ankle joint movement in response to mechanical stimulation with different intensities were investigated. The EMG analysis combined with ankle movement recording sought to determine the stimulation parameters for walking simulation, i.e., the intensities that were high enough to produce walking-like load sensation, but not high enough to activate reflex-induced ankle perturbation in terms of substantial change in the ankle joint.

## Methods

### Equipment description

**Figure 1:**
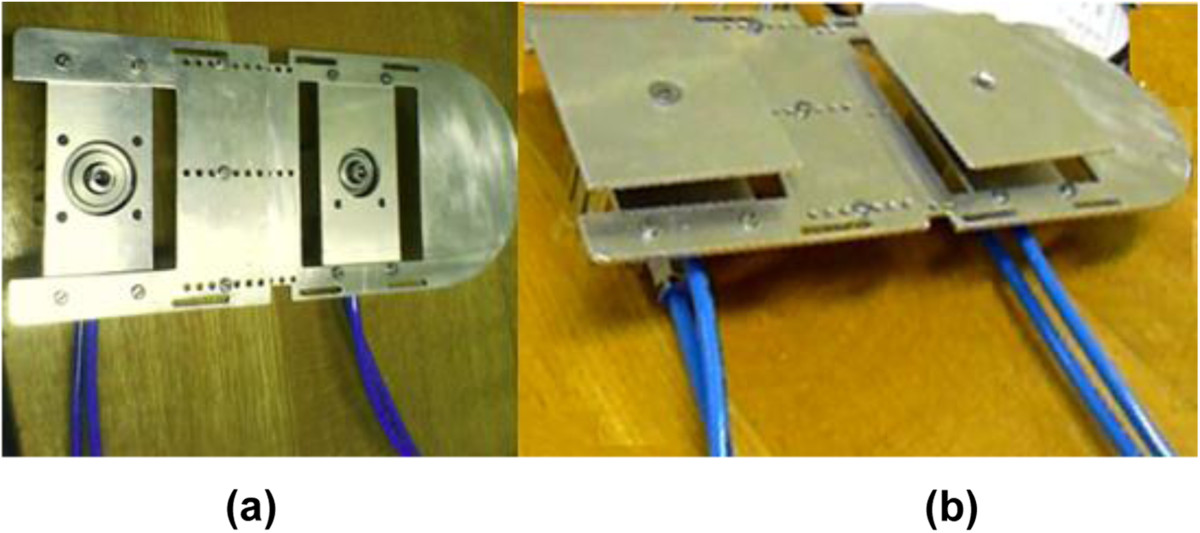
**The shoe platform structure. (a)** The foot plate (without pressure plates). **(b)** The two pressure plates rise for mechanical stimulation.

**Figure 2:**
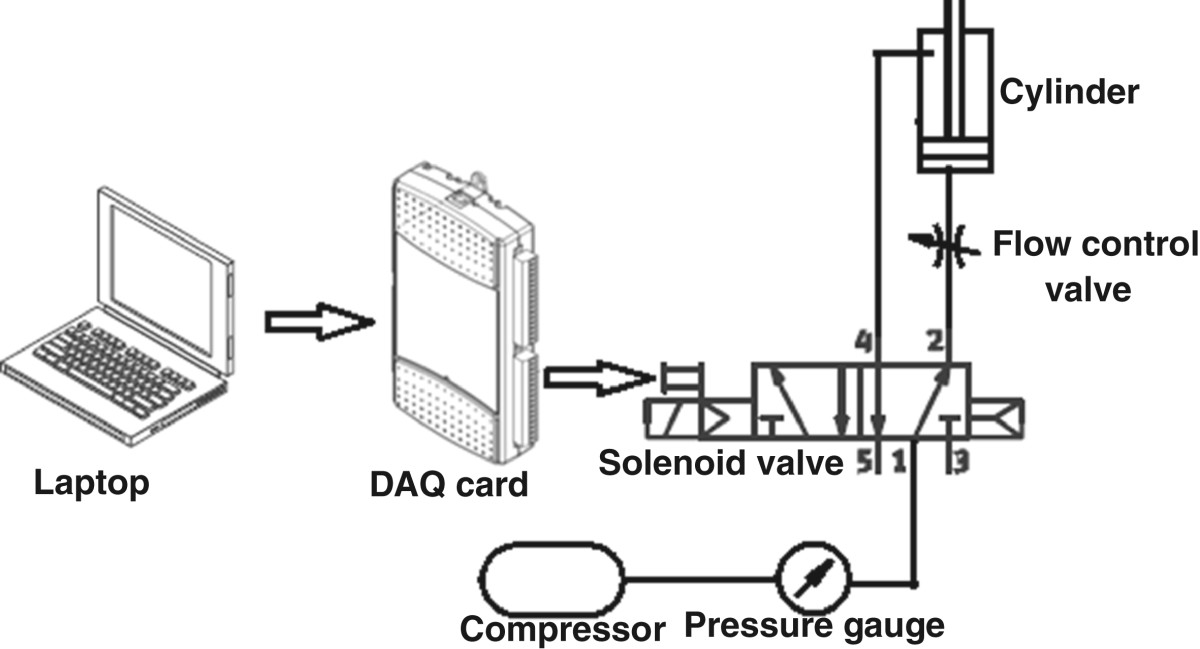
**The pneumatic system for mechanical force stimulation.**

With the aim of mimicking the upward ground reaction force, the shoe platform includes a foot plate and two pressure plates (Figure 1). The pressure plates for the heel (0.11 m × 0.08 m) and forefoot (0.11 m × 0.07 m) are actuated by cylinders (heel stroke 20 mm, diameter 32 mm; forefoot stroke 10 mm, diameter 25 mm). The cylinder in its neutral state is retracted, applying no loading on the foot sole. When activated by a solenoid valve, the retracted cylinder will extend fully (Figure 2) in 0.05 s, resulting in a fast upward movement of the pressure plate. Such upward stimulation in 0.05 s was defined as a fast stimulus. A one-way flow control valve regulates the rising speed of each pressure plate, to fully extend in 0.2 s. Such upward stimulation in 0.2 s was defined as a slow stimulus. Both pressure plates can be controlled independently, so that the platform can be adjusted to stimulate the foot sole with different patterns.

### Subjects and measurement devices

Ten able-bodied subjects were recruited (Table 1). Ethical approval was obtained from the Ethics Committee for Non Clinical Research, Faculty of Biomedical & Life Sciences, University of Glasgow. Subjects provided written informed consent prior to participation.

**Table 1:** **Subject information**

Subject	Age (yrs)	Gender	Mass (kg)	Height (m)	Foot length (m)	Forefoot width (m)	Hindfoot width (m)
S1	27	F	47	1.54	0.18	0.08	0.05
S2	24	M	53	1.6	0.2	0.09	0.06
S3	27	F	54	1.59	0.18	0.1	0.06
S4	28	F	56	1.62	0.18	0.09	0.05
S5	30	M	60	1.68	0.23	0.1	0.07
S6	27	M	72	1.7	0.23	0.1	0.06
S7	39	M	72	1.73	0.2	0.1	0.06
S8	28	M	72.5	1.82	0.24	0.11	0.07
S9	29	M	74	1.76	0.24	0.1	0.06
S10	24	M	88	1.94	0.25	0.1	0.08

**Figure 3:**
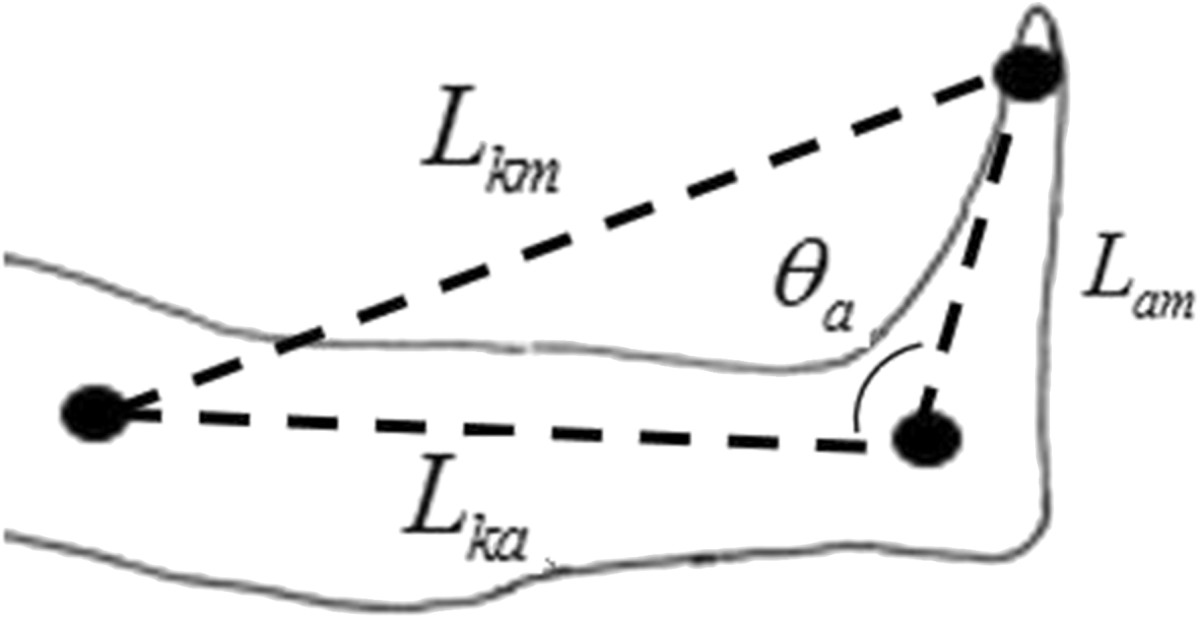
**Positions of Zebris markers for ankle movement recording.**

Bipolar EMG signals from the tibialis anterior (TA) and soleus (SOL) muscles were recorded by a GTEC amplifier (Guger technologies, Austria) via Matlab/Simulink (the MathWorks, Inc.). The sampling frequency of the EMG recording was 1200 Hz. An ultrasound system (zebris Medical GmbH, Allgäu, Germany) was employed to record foot motion at a frequency of 100 Hz. Three zebris markers were placed at the medial knee joint (*x*_*k*_, *y*_*k*_), the medial ankle joint (*x*_*a*_, *y*_*a*_) and the first metatarsal head (*x*_*m*_, *y*_*m*_) of the right leg (Figure 3). The ankle angle *θ*_*a*_, which was defined as the angle between the shank and the dorsum of the foot, was calculated as:

1 $$L_{\text{ka}}=\sqrt{{\left(x_{k}-x_{a}\right)}^{2}+{\left(y_{k}-y_{a}\right)}^{2}}$$

2 $$L_{\text{am}}=\sqrt{{\left(x_{a}-x_{m}\right)}^{2}+{\left(y_{a}-y_{m}\right)}^{2}}$$

3 $$L_{\text{km}}=\sqrt{{\left(x_{k}-x_{m}\right)}^{2}+{\left(y_{k}-y_{m}\right)}^{2}}$$

4 $$\theta_{a}=\pi +\text{arccos}\sqrt{\frac{{L_{\text{ka}}}^{2}+{L_{\text{am}}}^{2}-{L_{\text{km}}}^{2}}{2L_{\text{ka}}L_{\text{am}}}}$$

where *L*_*ka*_, *L*_*am*_ and *L*_*km*_ are the lengths between the knee and the ankle, the ankle and the first metatarsal head and the knee and the first metatarsal head, respectively. Reduction of the ankle angle corresponds to dorsiflexion and increase of this angle means plantarflexion.

### Test procedures

Before the test, the subjects took off their shoes and wore only their socks. They walked overground for several steps for two reasons: i) for the experimenters to confirm that the subjects had a normal gait pattern; ii) for the subjects to remember the sensation during overground walking, which later served as a reference when they provided their feedback on the shoe platform. Then each subject lay down on a mattress, with pillows inserted below the right knee joint. The dynamic shoe platform was fixed on the right foot. The ankle angle was approximately 150°. Minor adjustments were made to ensure that the subject lay comfortably on the mattress during the whole test.

The subject firstly performed maximal dorsiflexion and plantarflexion of the right foot three times to produce reference EMG signals during maximal voluntary contraction (MVC). The subject was then asked to lie relaxed. Four different pneumatic pressures were tested (Table 2). The maximum force was 280 N, which corresponds to approximately 30% to 60% of the recruited subjects’ body weight. The force range of 30% to 60% of body weight is similar to the force a patient usually experiences during treadmill training with body weight support [17].

**Table 2:** **Force amplitudes at various pneumatic pressures (manufacturer’s data** ^1^ **)**

Pressure (bar)	Force on the heel (N)	Force on the forefoot (N)
2	160	100
2.5	200	125
3	240	150
3.5	280	175

^**1**^Available from: http://www.festo.com/cms/en_corp/index.htm. Accessed on 20/06/2012.

The mechanical stimulation test included two sessions: single-pulse stimuli to determine the stimulation parameters and cyclic stimulation to simulate the walking-like loading.

#### Single-pulse stimuli

This session evaluated the influence of different parameters of the mechanical stimuli on the muscle response, including the rising speed of the pressure plate (slow and fast), location of the mechanical stimuli (the heel and the forefoot) and the pressure amplitude (2, 2.5, 3 and 3.5 bar). All of these parameters were combined, resulting in 16 types of stimuli taking place in a random order. Each type of stimulus was performed four times. Each stimulus was applied every 30 s and lasted for 0.8 s. The subjects had a 5-minute rest in the middle of the session.

#### Walking-like loading simulation

**Figure 4:**
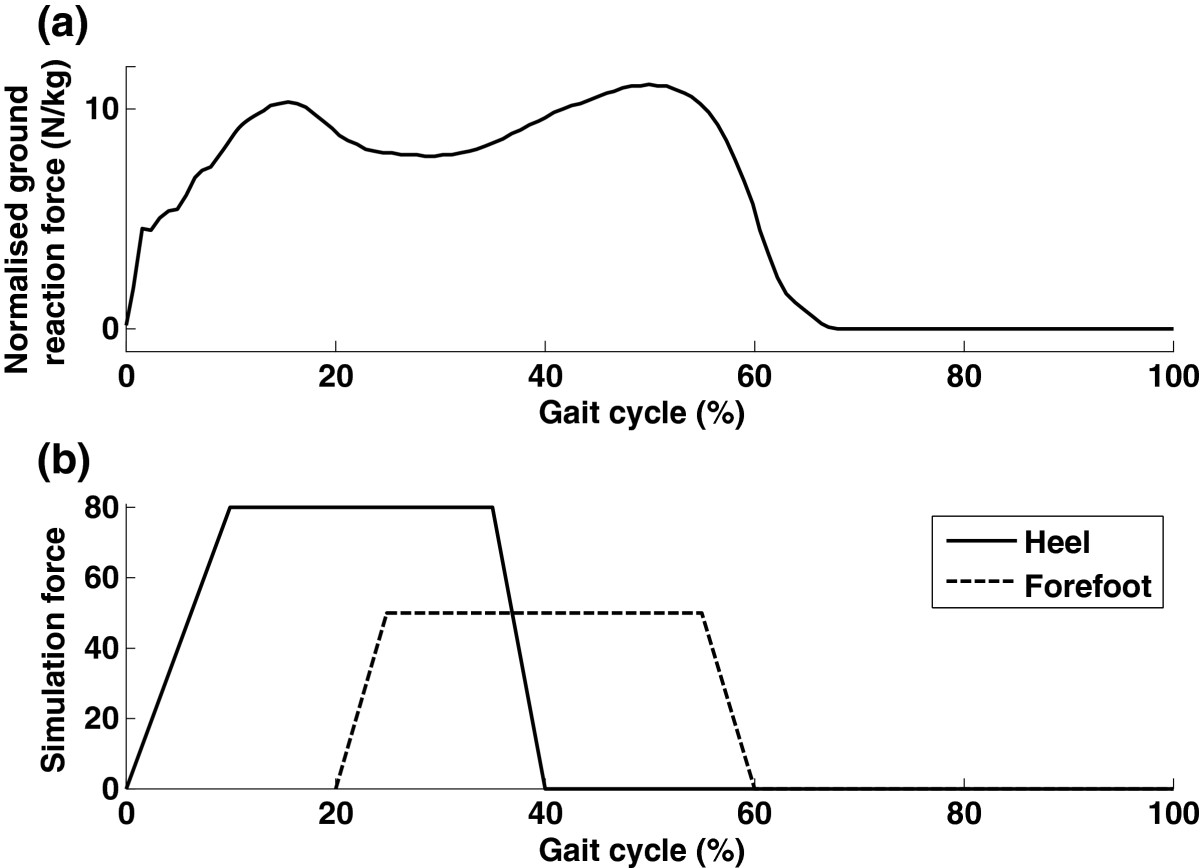
**Force patterns. (A)** a typical upward ground reaction force pattern during normal gait. The force shown here was the amplitude in Newtons normalized by body mass. This figure was adapted from [4]; **(B)**. The target force pattern produced by the shoe platform. The force shown here was the amplitude in Newtons at one bar.

During normal overground walking, the foot sole experiences ground reaction force within the stance phase, i.e., during 60% of the gait cycle. Peak forces occur around heel-strike and toe-off (Figure 4(a)). The simplified force pattern in Figure 4(b) was adopted to simulate the ground reaction forces that occur during walking. The simplified walking-like loads during short (2 s) and long (5 s) gait cycles were simulated. For example, to simulate the load occurring in a long gait cycle of 5 s, the heel and the forefoot were activated from 0–2 s and 1–3 s, respectively, in every 5 s interval. For a person with a height of 1.80 m and a step length of 0.85 m, these two selected gait cycles of 2 s and 5 s corresponded to walking speeds of about 3.0 and 1.2 km/h respectively, which are close to normally adopted walking speeds for patients during treadmill training [18]. The four pressures mentioned above (2, 2.5, 3 and 3.5 bar) combined with the two gait cycles resulted in 8 cyclic stimulation tests. Each test started with a 5-second rest, followed by 9 stimulation sequences (9 strikes). After the test, the subjects were asked the questions below so as to collect their feedback:

1. Are the locations of the pressure and the force timing (between heel and forefoot) similar to those during normal overground walking?
2. Is the walking-like stimulation (speed of force) on the heel and on the forefoot comparable to daily overground walking?
3. Do you feel comfortable with the dynamic force application?
4. In the case that you don’t have a walking-like feeling on your foot sole, please describe the main reason for this.
5. Choose the word that best describes your sensation during the mechanical force stimulation: A. Walking, B. Pressing, C. Punching, D. Jumping, E. None of these.

### Data analysis

EMG signals were recorded with a band-pass filter (5–500 Hz) and a notch filter (50 Hz), full-wave rectified and saved synchronously with the trigger signal for the pressure plate stimulation. The EMG data and foot motion recordings were visually observed to remove outliers. EMG data with high background noise were discarded. The foot motion data were further filtered with a window size of 5 to remove noise, and finally smoothed with the loess Matlab function.

For the EMG data during MVC, the RMS amplitude in a 500 ms time window centred at the maximal peak of the EMG signals was calculated [14, 19]. The maximal MVC_RMS_ (RMS EMG value during MVC) within the three repetitions was used as the reference for EMG normalisation.

For the EMG data from the single-pulse stimuli, the mean amplitude and standard deviation (SD) of the baseline raw EMG signals during the 0.8 s prior to stimulation were calculated and the reflex threshold was defined as mean + SD of the baseline EMG [20]. The reflex was deemed to have occurred if the EMG burst after the mechanical stimulation was larger than the threshold for a duration of 10 ms [20]. Different subjects had different reflex occurrences, which are expressed as a percentage. Each type of stimulus was repeated four times, therefore a reflex occurrence of 25% means that the reflex occurred once in four times and 100% means that the strong reflex occurred all four times.

In order to compare the EMG responses from slow and fast stimuli, the mean RMS values during the mechanical stimulation (0.8 s) at 3.5 bar of all subjects were calculated. Paired-sample one-sided t-tests were performed using SPSS (IBM Corp.) to determine whether the fast stimuli produced significantly higher EMG responses (significance level *p* = 0.05).

During the walking-like loading test, the mean RMS EMG amplitude during the total duration of the mechanical stimulation on the foot sole was calculated and compared to that of a 5 s pre-stimulation period, to investigate the EMG response from the stimulation.

## Results

RMS EMG values for all subjects at rest and during MVC are summarised below (Table 3). During the mechanical stimulation, reflexes with various amplitudes were observed in response to single-pulse stimuli and cyclic walking-load simulation. In the sequel, the EMG curves and the ankle angle traces from four representative subjects are presented in graphs to show typical EMG profiles induced by the mechanical stimuli, while the RMS EMG amplitudes and ankle angles of all the subjects under different pressures are presented as mean values in tables and bar plots.

**Table 3:** **RMS EMG values at rest and during MVC (μV)**

Subject	Rest		MVC	
	TA	SOL	TA	SOL
S1	1.16	1.11	162.81	126.52
S2	1.24	1.46	347.23	91.43
S3	1.86	1.52	211.12	54.08
S4	1.29	1.75	69.16	74.23
S5	1.07	1.03	255.32	31.28
S6	1.01	1.05	100.04	66.12
S7	1.15	1.58	220.45	115.57
S8	1.35	1.05	109.36	31.91
S9	1.04	1.18	97.78	50.26
S10	1.41	1.49	318.28	47.53
Mean ± SD	1.26 ± 0.25	1.32 ± 0.27	189.16 ± 97.33	68.89 ± 33.08

### Single-pulse stimuli

This test includes stimulation with the pressure plate rising slowly (slow stimuli) and quickly (fast stimuli).

#### (a) Slow stimuli

**Figure 5:**
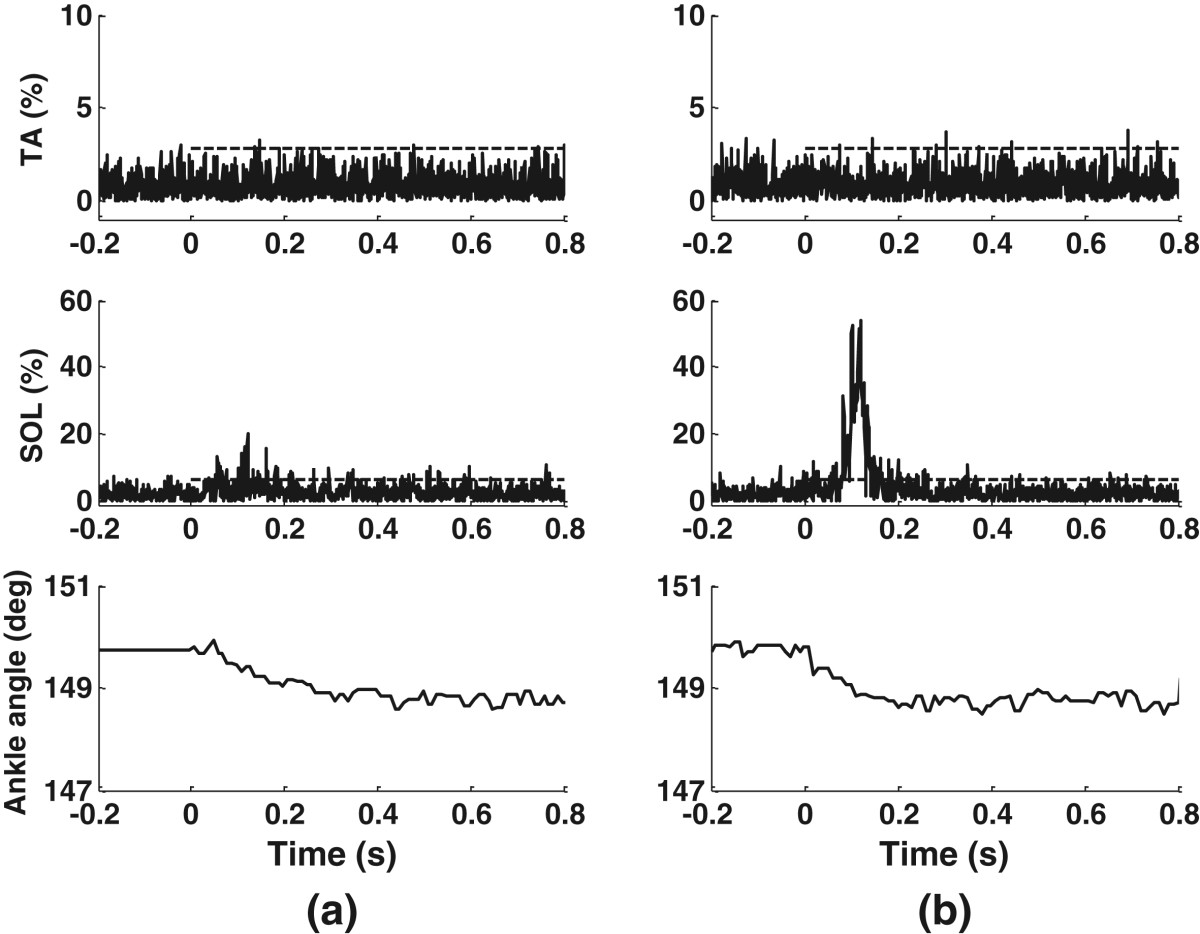
**Heel stimulation in S3.** The dashed lines mark the single-pulse mechanical stimulation periods, with the amplitudes of the dashed lines as the reflex thresholds. A larger EMG burst occurs in the SOL at higher pressure. **(a)**: 2 bar; **(b)**: 3.5 bar.

**Figure 6:**
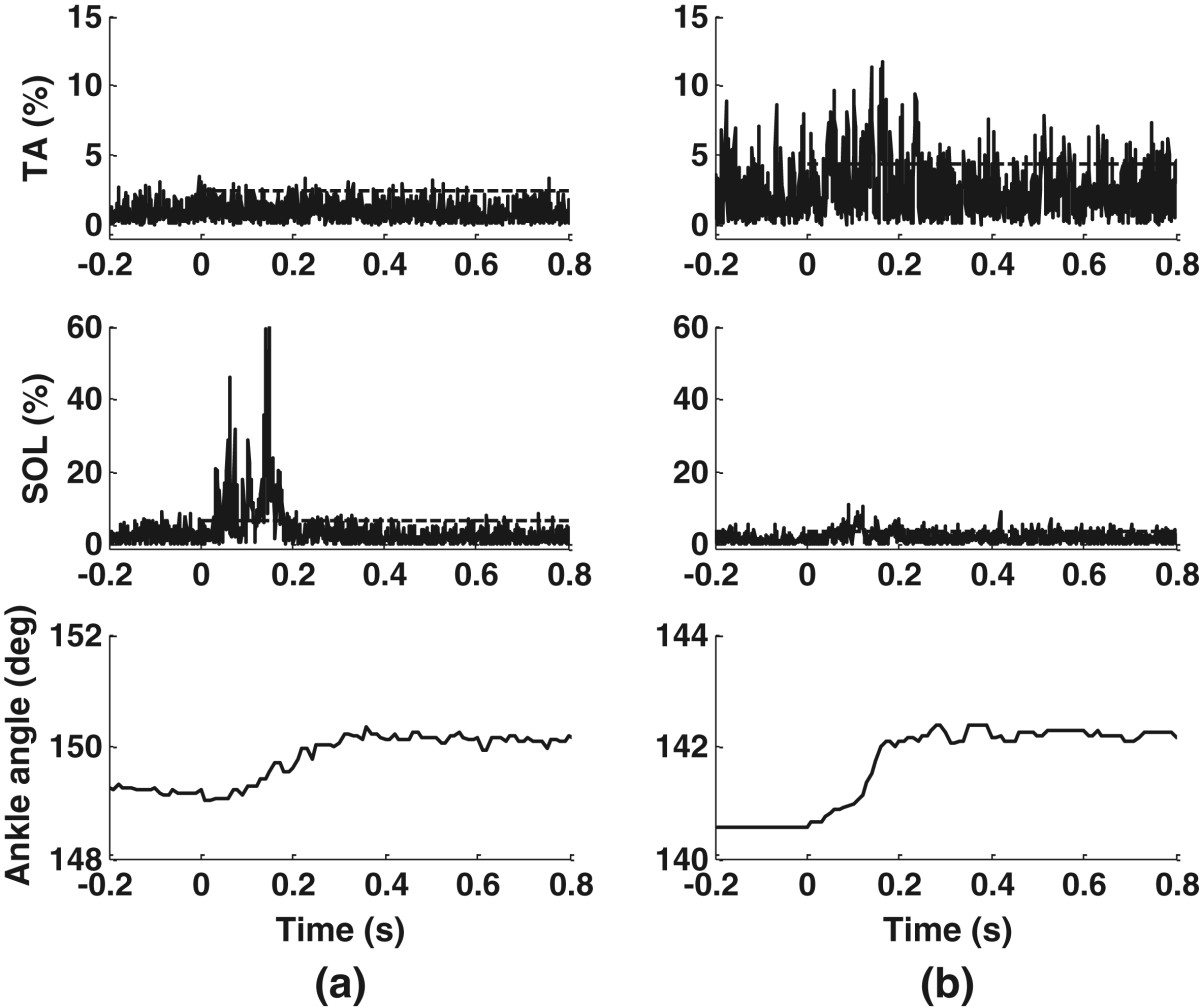
**Forefoot stimulation at 3.5 bar.** The dashed lines mark the single-pulse mechanical stimulation periods, with the amplitudes of the dashed lines as the reflex thresholds. Different subjects show different muscle activations. **(a)** S3; **(b)**: S4.

When the pressure plate took 0.2 s to reach full extension for mechanical stimulation, reflexes were observed in one or both of the lower leg muscles. Heel stimulation at 2 bar produced a weak reflex in the SOL (amplitude: 20.2% of MVC_RMS_) in subject S3 (Figure 5(a)). When the pressure increased to 3.5 bar, a larger EMG burst (amplitude: 54.5% of MVC_RMS_) was observed in the SOL (Figure 5(b)). The ankle angle of S3 gradually reduced by about 0.9° in response to heel stimulation, regardless of the pressure amplitude (the bottom plots in Figure 5).Forefoot stimulation induced EMG bursts in the same lower leg muscles as the heel stimulation, but the rise of the forefoot plate increased the ankle angle. For S3, forefoot stimulation at 3.5 bar produced a reflex in the SOL (Figure 6(a)) with a similar amplitude to that induced by heel stimulation at 3.5 bar (Figure 5(b)). Forefoot stimulation increased the ankle angle gradually by 0.8°. As well as EMG bursts in the SOL, some subjects showed TA activation from the mechanical stimulation. Subject S4 (Figure 6(b)) had both the TA and SOL activated by the forefoot stimulation.

**Figure 7:**
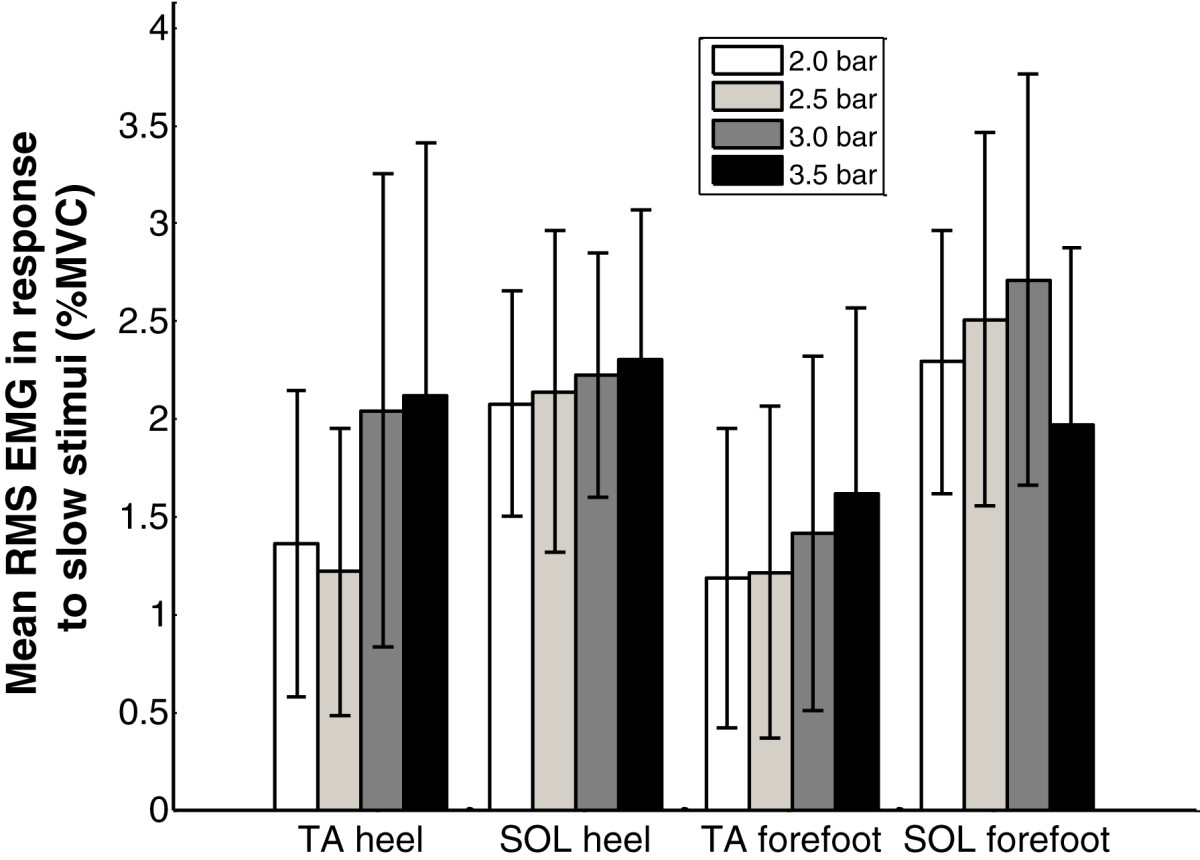
**The mean RMS EMG for all subjects induced by slow stimuli at various pressures.**

Slow stimuli on the foot sole produced limited reflexes in seven out of ten subjects (Table 4). Subjects S1, S3 and S7 had reflexes in the SOL only, S6 and S10 had increased muscle activity in the TA only, while S2 and S4 had EMG increases in both muscles. Subject S3 had the highest reflex occurrence in the SOL, while S4 had most reflex in the TA. Slow stimuli on the heel induced slightly more reflex activity than that on the forefoot (Table 4). The mean RMS EMG amplitudes during the four repetitions of slow stimuli at 3.5 bar for all subjects are presented in Table 5. It can be seen that all subjects had small RMS EMG amplitudes (less than 4.5% of MVC_RMS_). The change of the stimulation position from the heel to the forefoot was not found to change the muscle activated. The mean RMS EMG values during slow stimuli for all the subjects at four pressures are presented in Figure 7. It can be observed that higher forces generally produced a higher EMG response.

**Table 4:** **Reflex occurrence (%) for foot sole stimulation**

		Slow stimuli		Fast stimuli	
Subjects	Muscles	Heel	Forefoot	Heel	Forefoot
	TA	0	0	100	100
S1	SOL	25	0	100	100
	TA	0	25	25	50
S2	SOL	75	75	100	100
	TA	0	0	100	50
S3	SOL	100	100	50	25
	TA	75	50	100	100
S4	SOL	50	25	100	100
	TA	0	0	50	75
S5	SOL	0	0	100	25
	TA	50	25	100	100
S6	SOL	0	0	25	0
	TA	0	0	100	100
S7	SOL	25	0	100	100
	TA	0	0	50	0
S8	SOL	0	0	100	0
	TA	0	0	0	0
S9	SOL	0	0	0	0
	TA	25	0	100	100
S10	SOL	0	0	75	50

**Table 5:** **Mean RMS EMG values during slow stimuli at 3.5 bar (%MVC**_**RMS**_ **)**

Subject	Heel stimulation		Forefoot stimulation	
	TA	SOL	TA	SOL
S1	0.91	1.14	0.89	1.05
S2	0.46	3.12	0.89	2.30
S3	1.19	3.42	1.20	4.02
S4	3.21	2.77	2.48	2.21
S5	1.70	2.37	1.07	2.06
S6	4.41	2.02	3.26	2.10
S7	2.89	2.41	2.98	1.33
S8	1.51	2.73	1.53	2.38
S9	1.38	1.19	1.31	1.22
S10	3.50	1.85	0.52	1.01
Mean ± SD	2.12 ± 1.30	2.30 ± 0.76	1.61 ± 0.95	1.97 ± 0.90

The extension of the pressure plates applied forces on the foot sole, and also moved the ankle joint. During the mechanical stimulation, the heel or forefoot pressure plate dorsiflexed or plantarflexed the ankle joint, which reduced or increased the ankle angle, respectively (Table 6). The mean angle change of all the subjects was about 1°, irrespective of the pneumatic pressure.

**Table 6:** **The ankle angle change (degrees) induced by extension of the pressure plates during mechanical stimulation**

Subject	Heel stimulation	Forefoot stimulation
S1	-1.98	2.37
S2	-1.41	0.51
S3	-1.08	0.89
S4	-1.15	1.42
S5	-0.46	0.61
S6	-2.45	0.89
S7	-1.81	1.15
S8	-1.44	1.14
S9	-0.97	0.91
S10	-0.51	0.82
Mean ± SD	-1.33 ± 0.63	1.07 ± 0.53

#### (b) Fast stimuli

When the pressure plate was adjusted to achieve full extension in 0.05 s, reflexes were observed in nine out of ten subjects. Three of them showed reflex-induced ankle movements.

**Figure 8:**
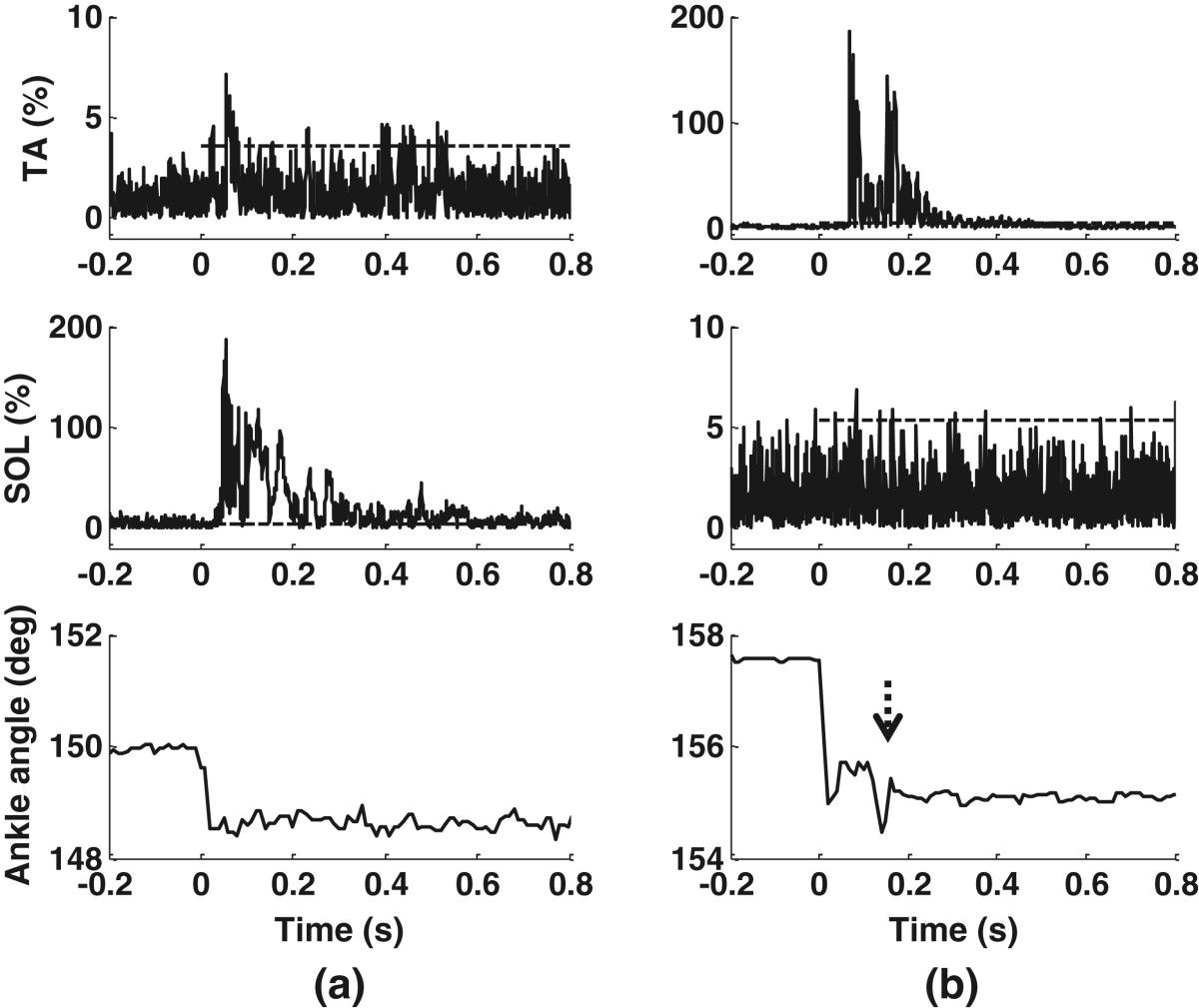
**The response to a fast stimulus on the heel.** The dashed lines mark the single-pulse mechanical stimulation periods, with the amplitudes of the dashed lines as the reflex thresholds. Fast stimuli increased the EMG amplitude. **(a)**: 2 bar in S3. **(b)**: 3.5 bar in S6. The dashed arrow shows reflex-induced ankle perturbation.

For S3, a fast stimulus at 2 bar induced a large EMG burst (amplitude: 186% of MVC_RMS_) in the SOL (Figure 8(a)). Furthermore, the TA was activated by the fast stimulus with an amplitude of 8% of MVC_RMS_. A higher pressure and a faster rising speed of the pressure plate both increased the EMG activity. Comparing Figures 8(a) and 5(b) with Figure 5(a) shows that an increased rising speed of the pressure plate had a larger influence on the muscle response.

Apart from the increased muscle activity, some subjects had additional ankle perturbations in response to a fast stimulus. In contrast to S3, Subject S6 readily had TA activation from the mechanical stimulation. Heel stimulation at 3.5 bar produced a strong reflex in the TA (amplitude: 197% of MVC_RMS_) with double bursts (Figure 8(b)). The heel pressure plate reduced the ankle angle by about 2.3° in S6. Furthermore, an additional change of 1° in the ankle angle (marked with a dashed arrow) was observed and was considered as an additional perturbation induced by the strong reflex. Such reflex-induced ankle movements were also observed in subjects S3 and S7.

**Figure 9:**
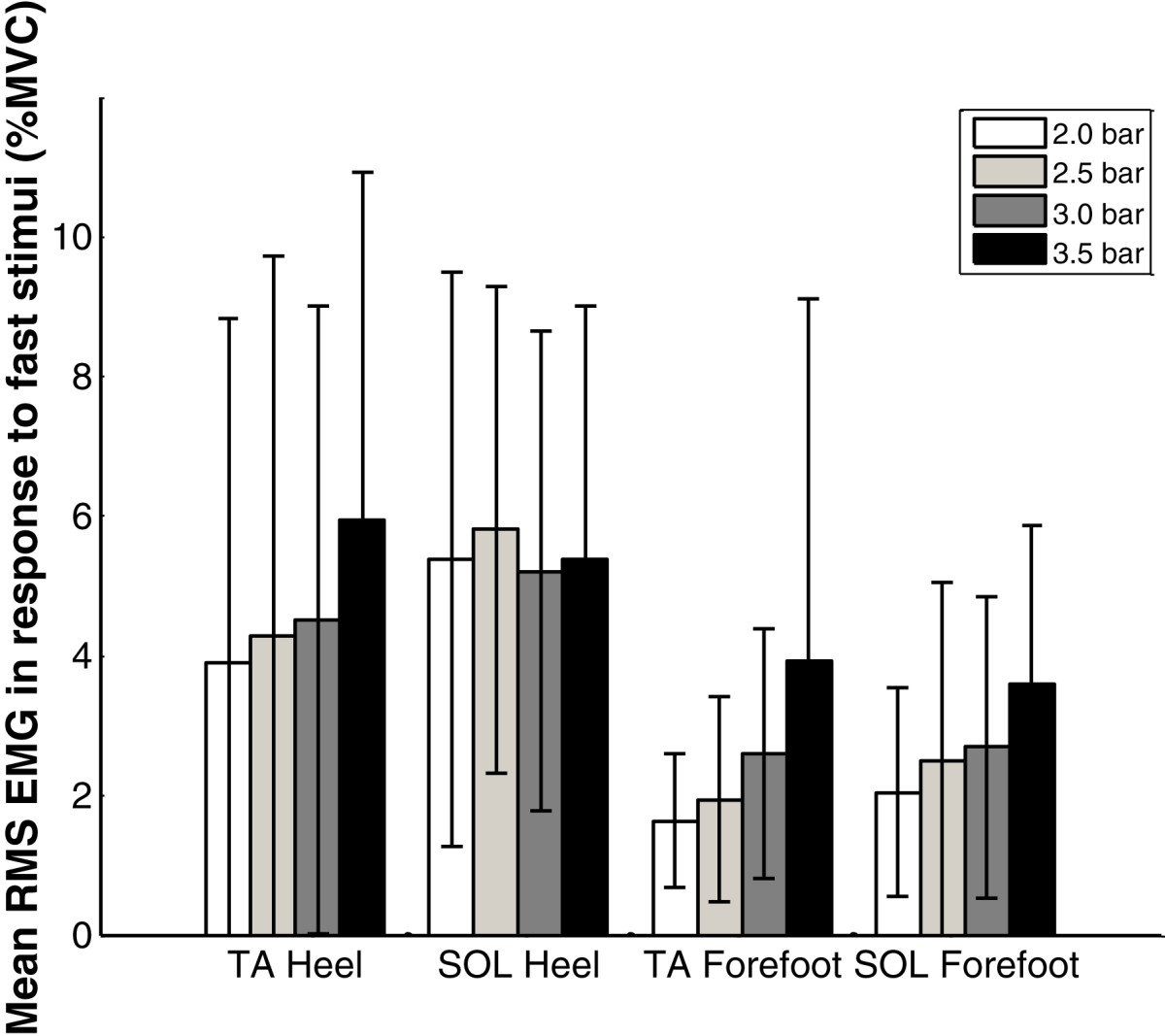
**The mean RMS EMG for all subjects induced by fast stimuli at various pressures.**

The reflex occurrence for all the subjects in response to fast stimuli at 3.5 bar are also presented in Table 4. Similar to slow stimuli, fast stimuli on the heel brought more reflex activity than on the forefoot and TA was more easily activated than SOL. However, fast stimuli increased the reflex occurrence. Subjects S1, S4 and S7 had reflexes for every fast stimulus. The mean RMS EMG values during fast stimuli for all the subjects at four pressures are presented in Figure 9. It can be observed that higher forces generally produced a higher EMG response.

The mean RMS EMG amplitudes during fast stimuli at 3.5 bar for all subjects are presented in Table 7. Compared to slow stimuli (Table 5), much higher RMS EMG amplitudes were observed if the pressure plate rose quickly, with the maximal RMS amplitude up to 17% and 13% of MVC_RMS_ in the TA and the SOL, respectively. Pairwise comparisons were carried out on the four experimental conditions reported in Tables 5 and 7 to determine whether the fast stimuli produced higher mean EMG responses. For this analysis, the four conditions tested (Heel-TA, Heel-SOL, Forefoot-TA, Forefoot-SOL) were considered as separate experiments. P-values from paired-sample one-sided t-tests are shown for each condition in the bottom row of Table 7: for three of the conditions (Heel-TA, Heel-SOL and Forefoot-SOL), the fast stimuli produced significantly higher EMG responses while the remaining condition (Forefoot-TA) showed a trend for a higher EMG response.

**Table 7:** **Mean RMS EMG values during fast stimuli at 3.5 bar (%MVC**_**RMS**_ **)**

Subject	Heel stimulation		Forefoot stimulation	
	TA	SOL	TA	SOL
S1	1.17	2.11	8.82	6.58
S2	0.49	6.68	0.46	2.63
S3	3.52	13.34	1.32	8.02
S4	7.86	6.75	3.31	2.65
S5	6.52	3.23	2.88	4.61
S6	13.78	2.16	17.00	1.96
S7	11.67	4.83	1.58	1.95
S8	1.73	4.87	1.65	4.39
S9	1.47	1.27	1.45	1.29
S10	11.19	8.36	0.67	1.88
Mean ± SD	5.94 ± 4.97	5.36 ± 3.64	3.91 ± 5.19	3.60 ± 2.25
p-values	0.0054	0.0064	0.0808	0.0117

P-values are for comparisons with means in Table 4.

### Walking-like loading simulation

As the reflex-induced ankle movements observed during fast stimuli should be avoided during walking training, the stimulation pattern with slow stimuli, i.e., the pressure plate fully extended in 0.2 s, was used to simulate walking-like loading.

**Figure 10:**
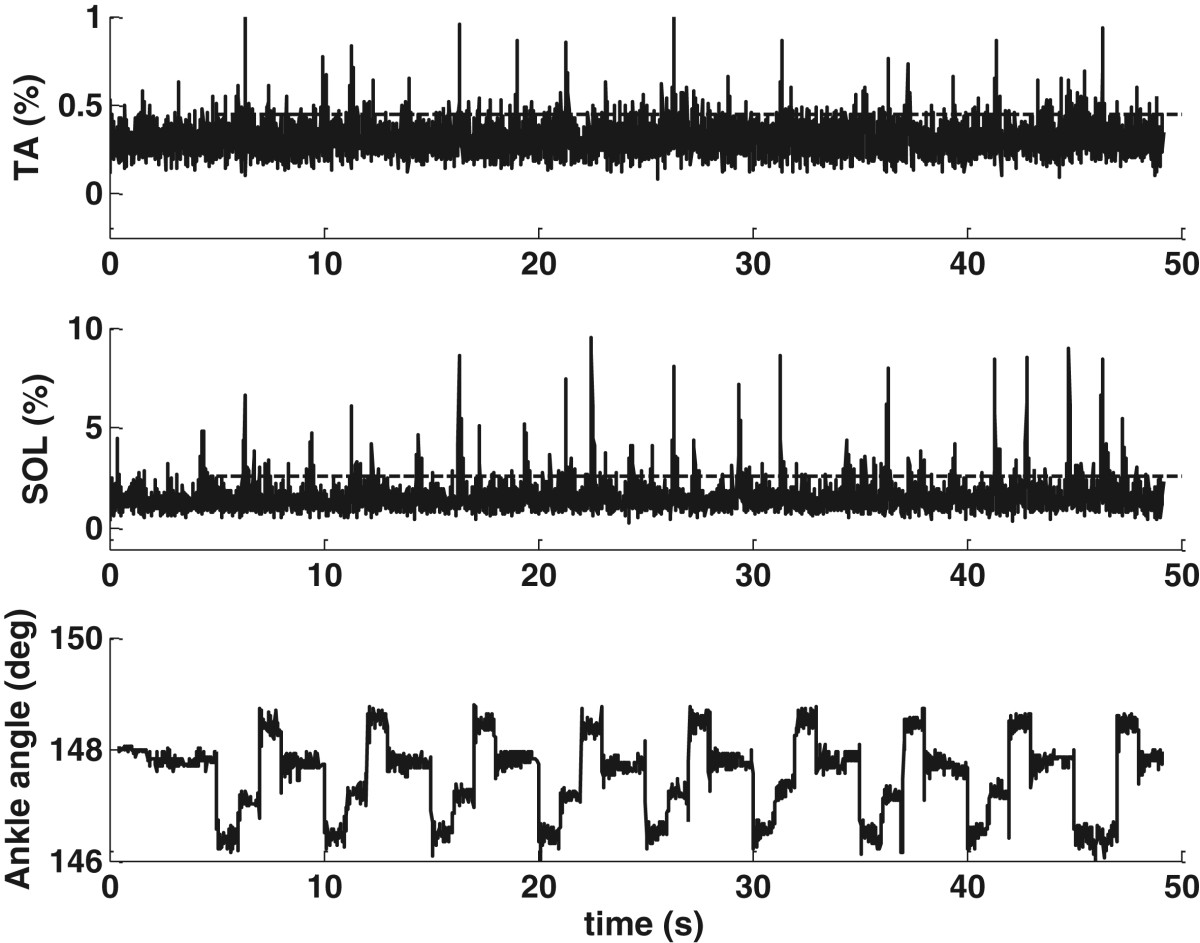
**Walking-like load simulation for the long gait cycle at 2 bar in S2.** The EMG data are resampled at 100 Hz to make the EMG profiles easier to discern.

**Figure 11:**
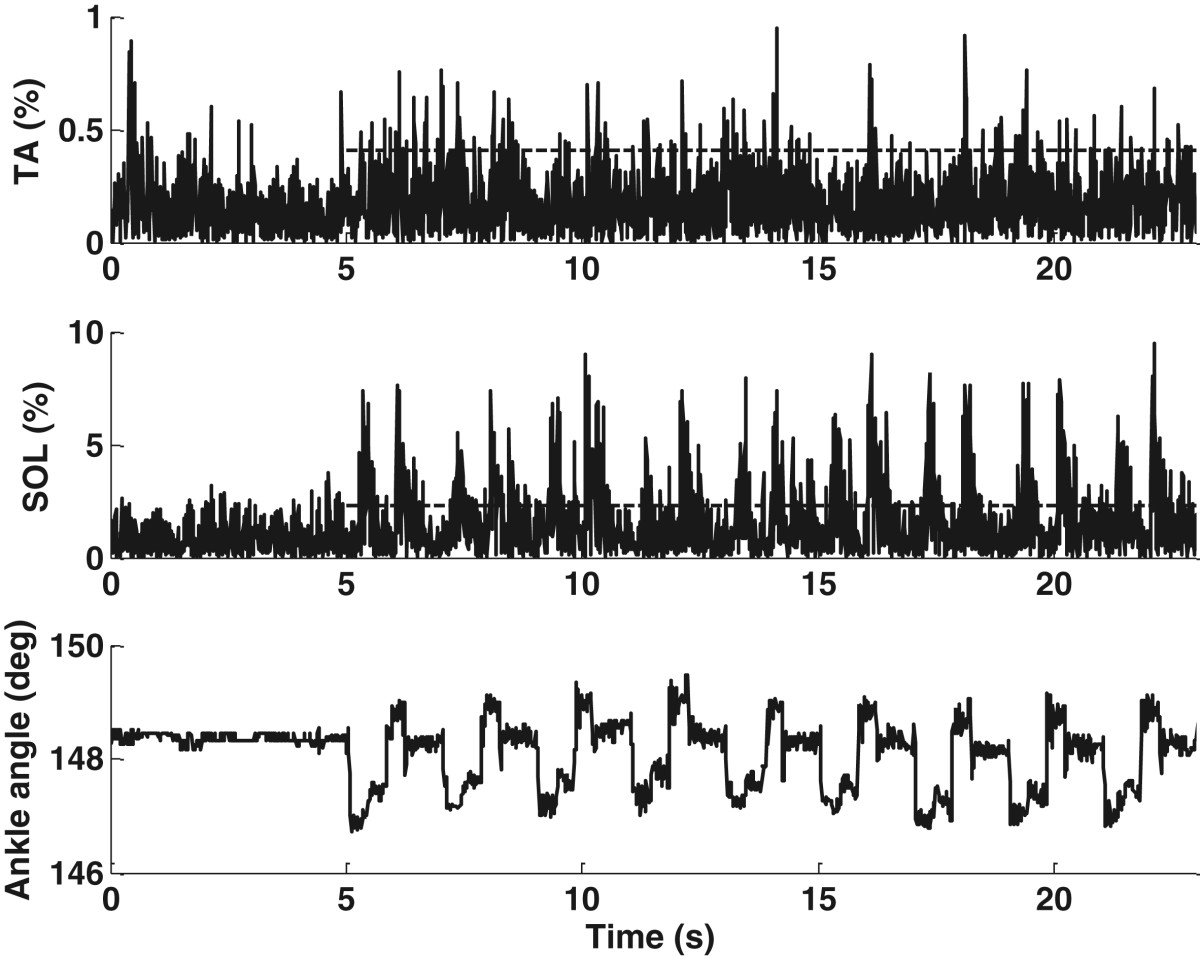
**Walking-like load simulation for the short gait cycle at 3.5 bar in S2.** The EMG data are resampled at 100 Hz to make the EMG profiles easier to discern. EMG bursts are observed in response to cyclic force stimulation.

**Figure 12:**
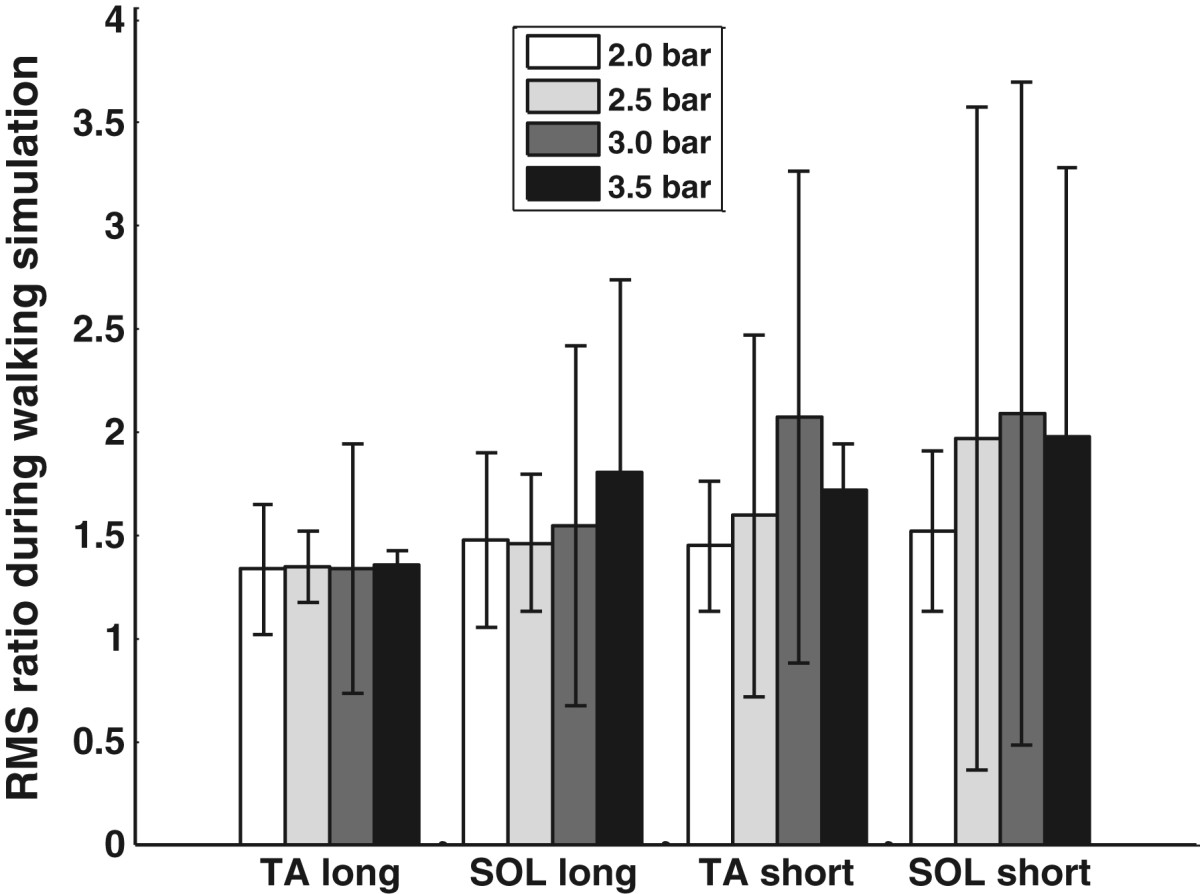
**RMS EMG values (relative to resting state) during walking-like load simulation.** “Long” and “short” refer to the long gait cycle of 5 s and the short gait cycle of 2 s.

As expected, mechanical stimulation with the walking-like loading patterns increased the muscle activity, with the EMG responses of representative subject S2 shown in Figures 10 and 11. Simulation of the loading during the long gait cycle, even at 2 bar (Figure 10), gave observable EMG bursts in the SOL. The simulation for the short gait cycle, even at 3.5 bar (Figure 11), increased the EMG without strong reflexes or reflex-induced ankle movement. The SOL had increased EMG corresponding to the rise of each pressure plate. The ankle angle changes induced by the cyclic mechanical stimulation were about 1.3° from heel stimulation and about 0.7° from forefoot stimulation in S2 (the bottom plots in Figures 10 and 11). These results were similar to those during the single-pulse stimuli shown in Table 6.Nine of the ten subjects had increased muscle activity during stimulation, compared to the resting situation. The mean RMS EMG amplitudes during walking-like loading simulation at variable pressures relative to the resting state for the nine subjects are presented in Figure 12. It can be seen that a higher pressure induced a larger EMG response, with the largest amplitude occurring in the SOL during the simulation of the load for the short gait cycle, and the smallest amplitudes observed in the TA during the simulation of the long gait cycle.

All ten subjects felt cyclic force patterns on the foot sole and reported a stronger loading sensation if a higher pressure was applied. Nine subjects thought the shoe platform had the right location of stimulation for walking simulation, while one subject S6 thought the force on the top of the foot caused by the Velcro straps made the feeling different from walking. Subjects 1–4, 7 and 8 thought the stimulation had force timing similar to walking. Among the four subjects who perceived timings different from walking, Subjects 6 and 10 thought the delay between the heel and forefoot stimulation was too long during the simulation of the load for the long gait cycle, while Subjects 5 and 9 found it hard to express why they felt different. Subjects 1–6 and 8 considered the rising speed of force on both the heel and the forefoot to be similar to overground walking; Subjects 7 and 10 thought the force on the heel had a better feeling than that on the forefoot, while one subject S9 thought the force on the forefoot felt better. Seven subjects thought the shoe platform was comfortable to use while Subjects 2, 9 and 10 were neutral (neither comfortable nor uncomfortable). Subjects 1 and 3–7 described the stimulation as pressing, Subjects 8 and 10 described it as punching and Subjects 2 and 9 described it as walking.

## Discussion

The aim of this work was to investigate the EMG and the ankle angle responses induced by mechanical stimulation from the pneumatic shoe platform, thereby evaluating the performance of the shoe platform as a rehabilitation tool for simulation of walking-like forces on the foot sole of users in a supine position.

The shoe platform included two pressure plates to produce adjustable forces on the heel and the forefoot with an adaptable timing. The simplified force pattern (Figure 4(b)) had similar stimulation locations to the normal force pattern (Figure 4(a)). The shoe platform had similar stimulation timings to the normal force pattern: the heel had mechanical stimulation for 40% of the gait cycle, and the forefoot was stimulated during 20-60% of the gait cycle. The heel and the forefoot were stimulated together for 20% of the gait cycle to simulate the mid-stance phase. However, in contrast to the force pattern during overground walking (Figure 4(a)), where force amplitudes around heel-strike and toe-off were very close, our shoe platform produced a larger force on the heel than on the forefoot, because the area of the pressure plate on the heel was larger than on the forefoot, resulting in a requirement of a larger force on the heel to achieve a similar pressure to that on the forefoot. Furthermore, the heel is not as sensitive to stimulation as the forefoot [16]. We selected a larger force for the heel for simulating the heel-strike shock [21]. In summary, the target force patterns of the shoe platform had similar stimulation locations and force timings to those during real overground walking. The force amplitude was different, with the aim to simulate the heel-strike dynamics.

Comparing the responses from the slow and fast stimuli, we found that the fast rising speed of the pressure plate increased the EMG values but with the possibility of inducing ankle perturbation. Among the stimulation parameters, the stimulation location was not found to affect the activated muscle. Depending on the subject, the foot stimulation activated one or both of the lower leg muscles studied, regardless of whether the heel or the forefoot was stimulated. The muscle activity had a small increase in response to an increase in the force amplitude, but had a significant increase in response to an increased force application rate. Fast stimuli produced double-burst reflexes with additional ankle perturbations (Figure 8(b)), which agrees with a previous description of withdrawal reflexes [22]. As our study was not designed to investigate the reflex patterns in response to foot sole stimulation, our experiment was not arranged to use the platform to target specific types of receptors. The shoe platform might stimulate the cutaneous mechanoreceptors by dynamic forces, resulting in cutaneous reflexes. The movement of the pressure plate changed the ankle angle, which might bring a stretch reflex. Fast stimuli might also produce withdrawal reflexes. The origin of the reflexes obtained here was not the focus of this study and requires further investigation. From this study we obtained the parameters which avoid the strong reflexes, which meets the aim of our study.

The shoe platform, as a component in the gait orthosis, should avoid these reflex-induced movements to ensure the safety of the user. Therefore, during the walking-like loading simulation, the shoe platform stimulated the foot sole slowly, with the pressure plate extending fully in 0.2 s. By controlling the supply pressures and pneumatic valves, the shoe platform produced various force amplitudes on the foot sole, which allowed simulation of the ground reaction forces that occur during walking at different speeds. The load simulation for both long and short gait cycles increased the EMG activity in the lower leg muscles. This observation is consistent with a previous study which showed that foot loading increases the EMG in the TA during air stepping [23]. A higher pressure produced stimulation with a higher intensity, resulting in a larger EMG response. However, the loading simulation at 3.5 bar did not produce reflex-induced ankle perturbation, which avoids the risk of injury to the user.

Muscle activity, to some extent, reflected the stimulation intensity, which is believed to be related to the sensation intensity for a subject with a normal sensorimotor system. All the subjects felt mechanical stimulation on the foot sole, with the sensation becoming stronger with the pneumatic pressure. Nine subjects showed corresponding increases in the EMG bursts from the TA and/or SOL. The EMG from the gastrocnemius muscle was not recorded in this study, because in a supine position this muscle contacts the bed. The mechanical loading induced some leg motion and produced friction between this muscle and the bed, which might have interfered with the EMG recordings on the gastrocnemius. However, the gastrocnemius muscle can also absorb part of the applied mechanical load. This might explain why TA/SOL muscle activation was not observed in many cases. Among the ten subjects, six subjects thought the mechanical stimulation had similar force timings to walking. Seven subjects anecdotally reported that the rising speed of force on both the heel and the forefoot to be similar to overground walking. Although this feedback was subjective, they provide typical assessments of the shoe platform [12].

The force rising time was found to be an important issue to consider during simulation of the ground reaction force. The rising time of the ground pressure ranges from 0.08 s to 0.16 s during overground walking at normal cadence [2]. When the walking speed slowed to 75% of normal cadence, the rising time prolonged to about 0.25 s [2]. In our study, we found that stimulation force with a rising time of 0.05 s produced strong reflexes. To prevent this, we prolonged the rising time to 0.2 s, because i) the pneumatic insole [12] took about 0.2 s for simulation of ground force, which serves as a reference parameter for our study; ii) we aimed to simulate normal and slow walking, therefore we adopted a rising time in the middle of the range (0.08-0.25 s) of the actual rising time during walking at various speed. To ensure test accuracy, the control valve was adjusted to the target position of 0.2 s and kept at this level during the whole test. A digital valve to accurately control the force rising time is required so that stimulation with other rising times, such as 0.1 s, can be investigated.

The limitation of the shoe platform is that the extension of the pressure plate for mechanical stimulation changed the ankle angle by about 1° on average (Table 6), which represents about 3% of the range of motion of the ankle joint during normal gait [21]. This ankle movement is unavoidable for the current platform structure, but can be reduced if cylinders with a shorter stroke are used.Further work is required to improve the shoe platform. Pressure sensors should be inserted between the pressure plates and the foot sole so as to record how much force is actually applied on the foot by the shoe platform. The force pattern should be refined by adopting a pressure control valve to adjust the pressure. Although most subjects reported that the stimulation profile (Figure 4(b)) was similar to the ground reaction forces that occur during overground walking, the shoe platform needs to control the retraction speed of the pressure plates for better simulation of walking-like loading. The pneumatic system was easy to control, but noise should be reduced by adopting noise silencers. Further tests should be carried out in neurological patients so as to investigate the potential for clinical application.

## Conclusions

The study determined the stimulation parameters and demonstrated the technical feasibility of the dynamic shoe platform to simulate walking-like forces on the foot sole. With able-bodied users in a supine position, the shoe platform applied mechanical forces on the foot sole, which increased the EMG bursts in the lower leg muscles and produced loading sensation that is similar to the ground reaction forces during overground walking. The shoe platform was demonstrated to be a useful tool for stimulation of the foot sole, thus it has potential to be incorporated in a gait orthosis for ground reaction force simulation.

## Authors’ original submitted files for images

Below are the links to the authors’ original submitted files for images. Authors’ original file for figure 1 Authors’ original file for figure 2 Authors’ original file for figure 3 Authors’ original file for figure 4 Authors’ original file for figure 5 Authors’ original file for figure 6 Authors’ original file for figure 7 Authors’ original file for figure 8 Authors’ original file for figure 9 Authors’ original file for figure 10 Authors’ original file for figure 11 Authors’ original file for figure 12
