# Supplementary figures and images for: Mechanical stimulation of the foot sole in a supine position for ground reaction force simulation
Source: J Neuroeng Rehabil. 2014 Nov 28;11:159. doi: 10.1186/1743-0003-11-159 (PMC4280696; doi:10.1186/1743-0003-11-159)

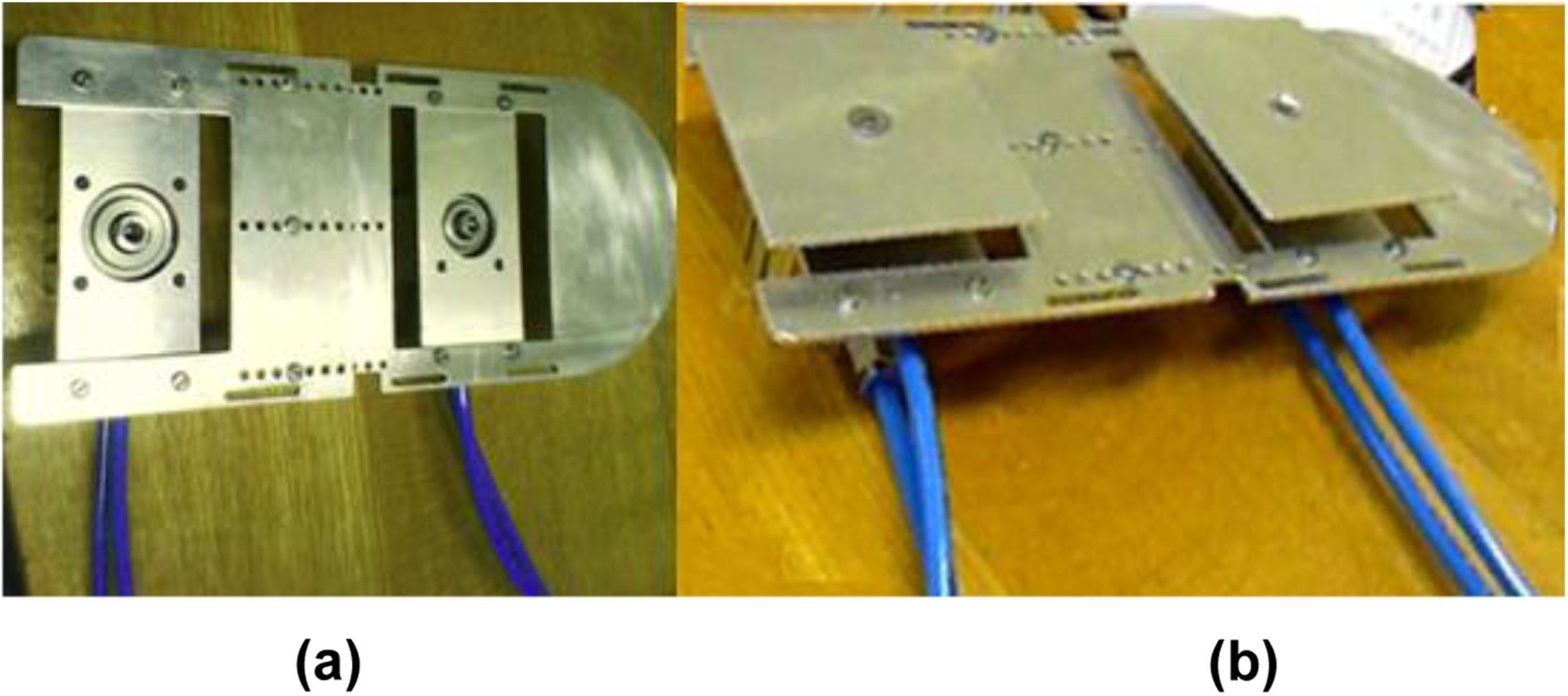

Supplement: Supplementary file 1 — Authors’ original file for figure 1 [file 12984_2014_688_MOESM1_ESM.tif]

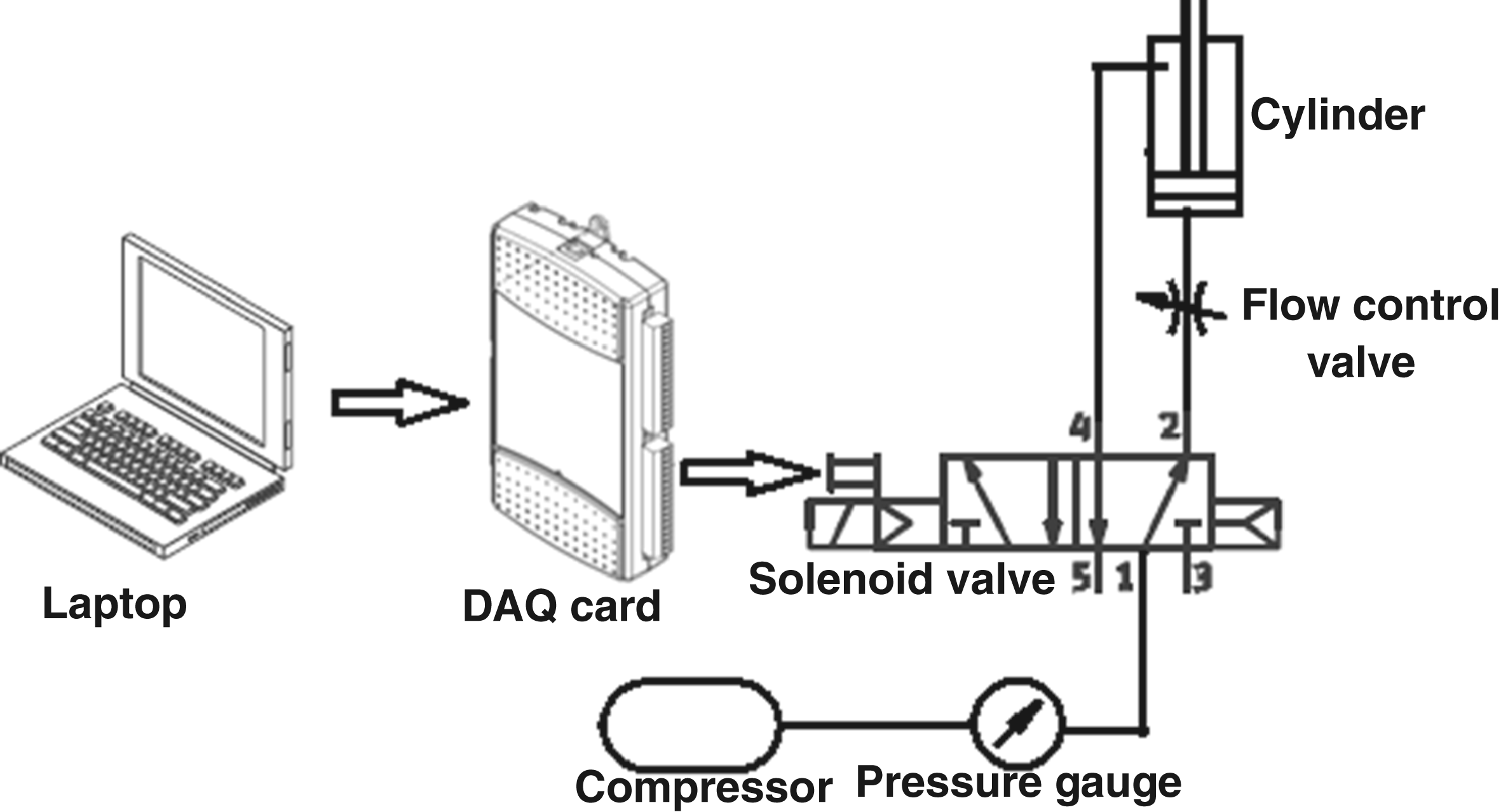

Supplement: Supplementary file 2 — Authors’ original file for figure 2 [file 12984_2014_688_MOESM2_ESM.tif]

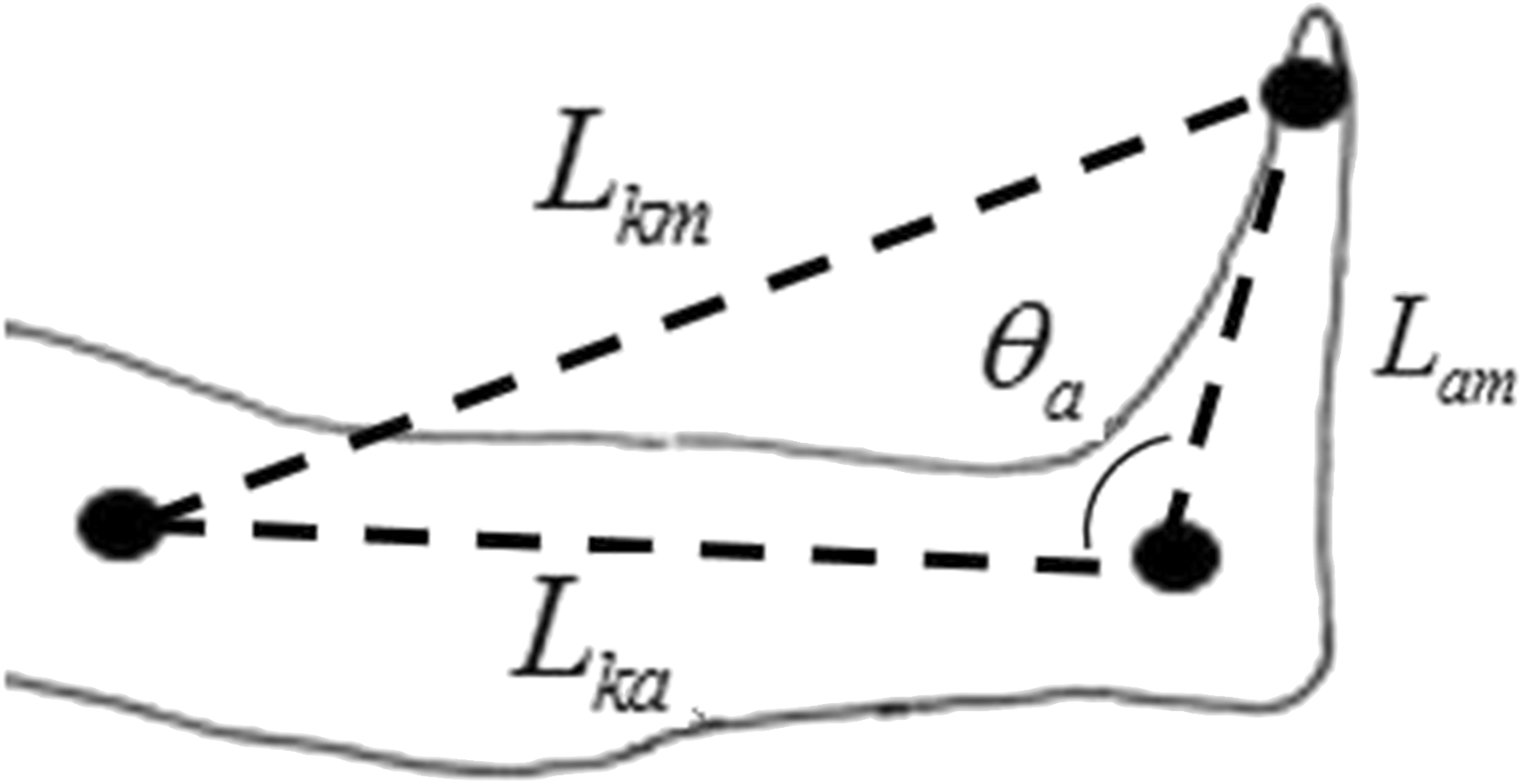

Supplement: Supplementary file 3 — Authors’ original file for figure 3 [file 12984_2014_688_MOESM3_ESM.tif]

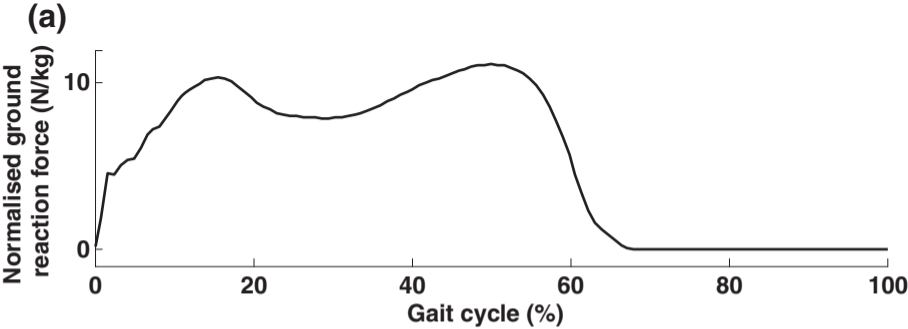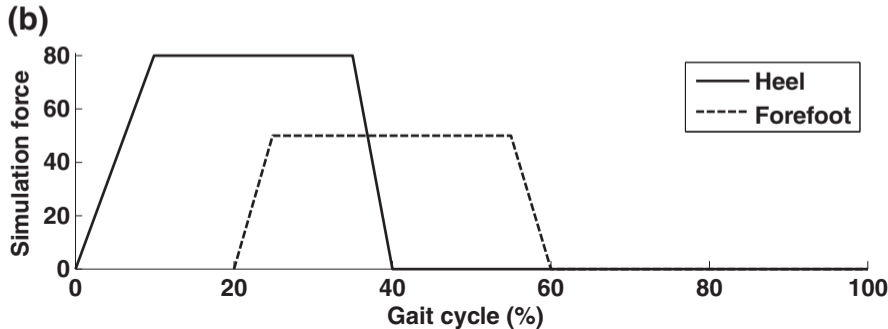

Supplement: Supplementary file 4 — Authors’ original file for figure 4 [file 12984_2014_688_MOESM4_ESM.pdf]

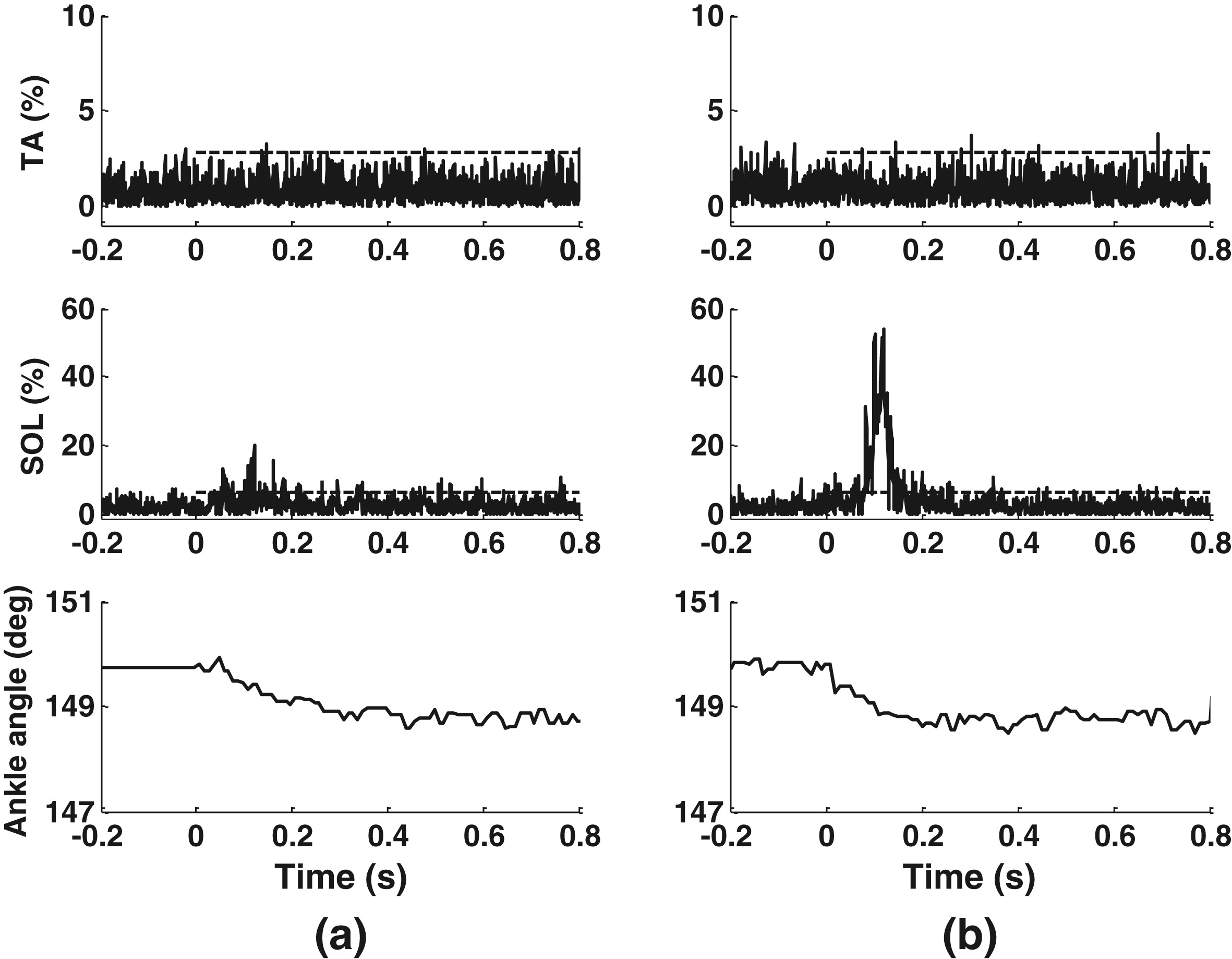

Supplement: Supplementary file 5 — Authors’ original file for figure 5 [file 12984_2014_688_MOESM5_ESM.tif]

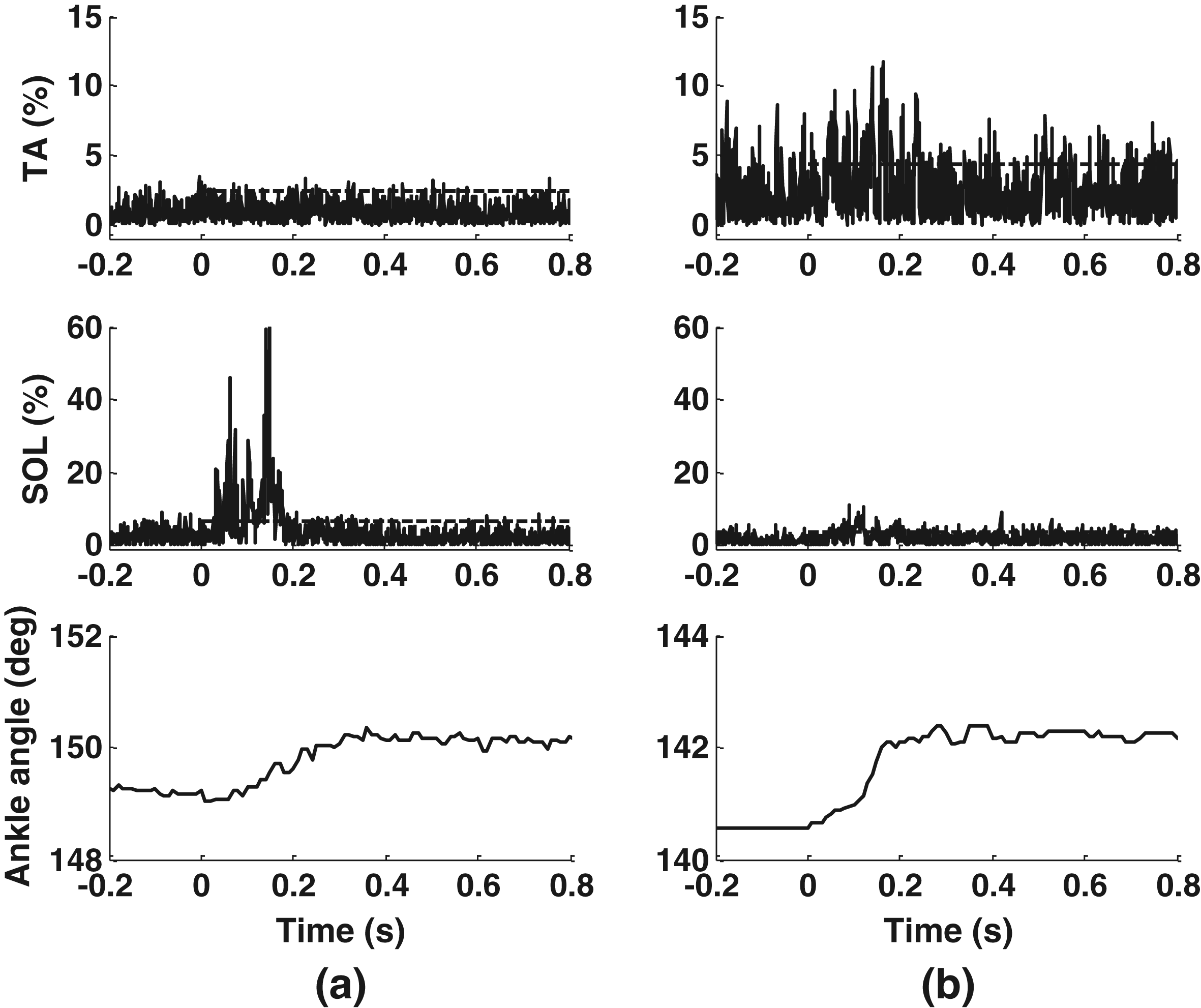

Supplement: Supplementary file 6 — Authors’ original file for figure 6 [file 12984_2014_688_MOESM6_ESM.tif]

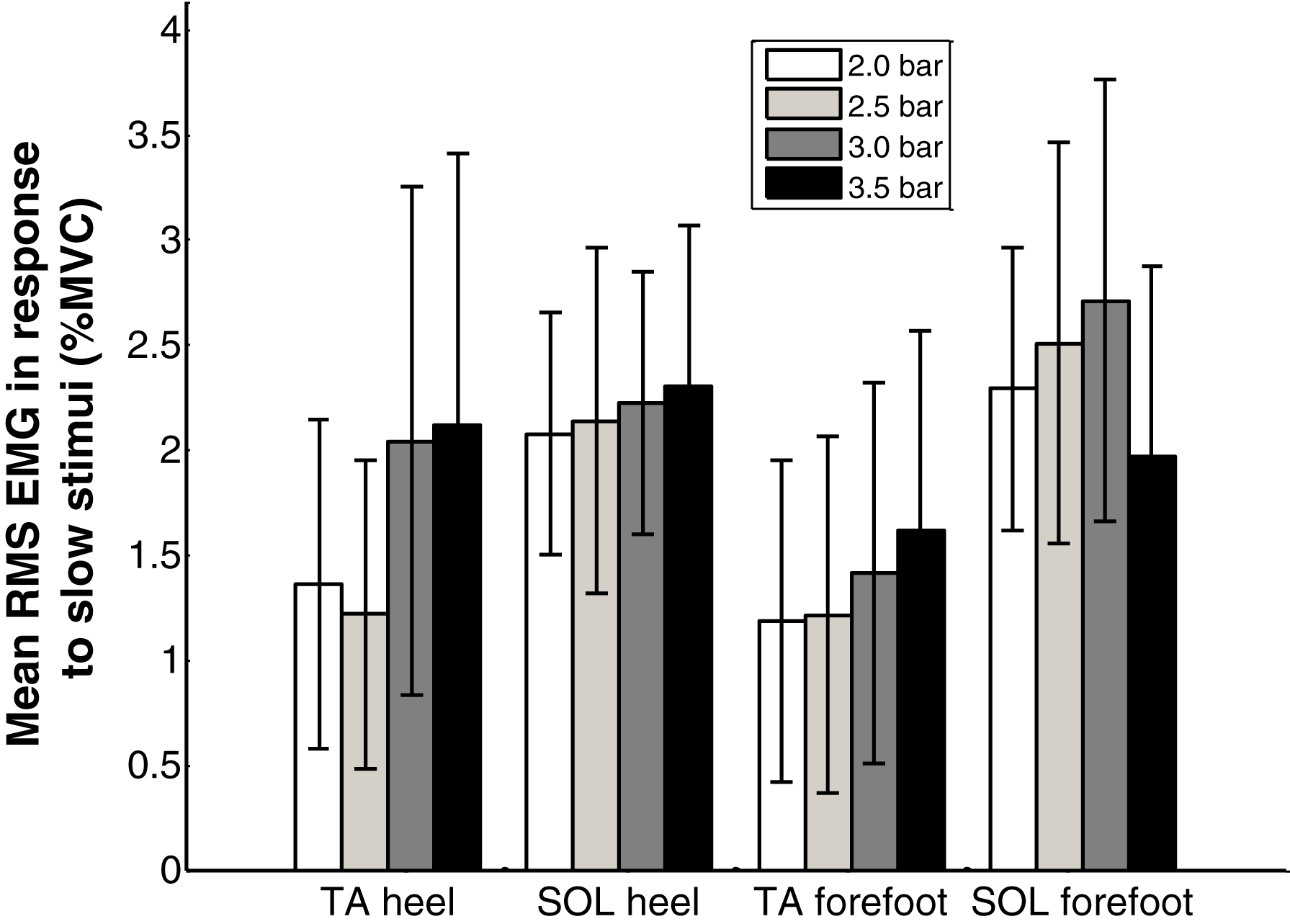

Supplement: Supplementary file 7 — Authors’ original file for figure 7 [file 12984_2014_688_MOESM7_ESM.tif]

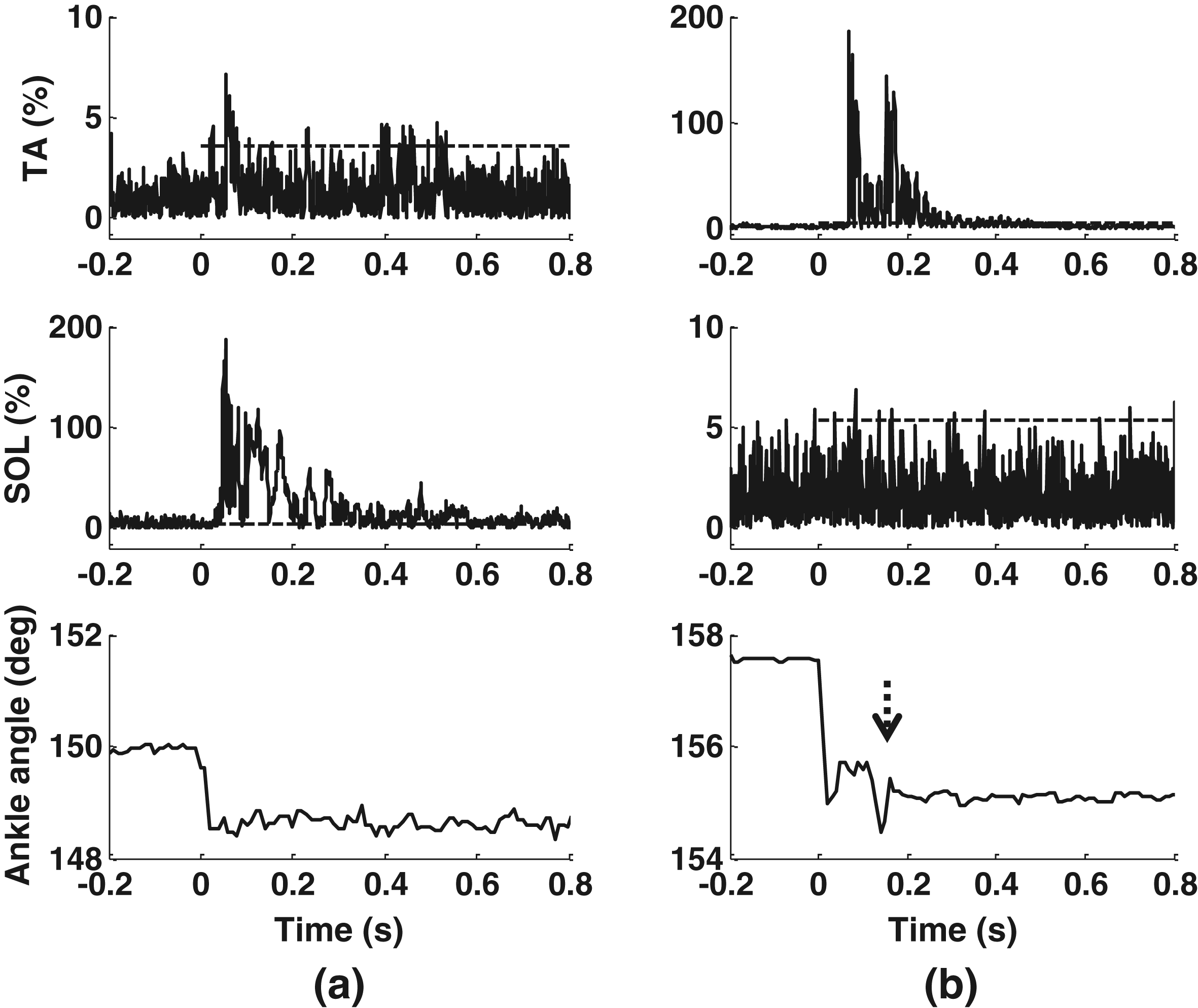

Supplement: Supplementary file 8 — Authors’ original file for figure 8 [file 12984_2014_688_MOESM8_ESM.tif]

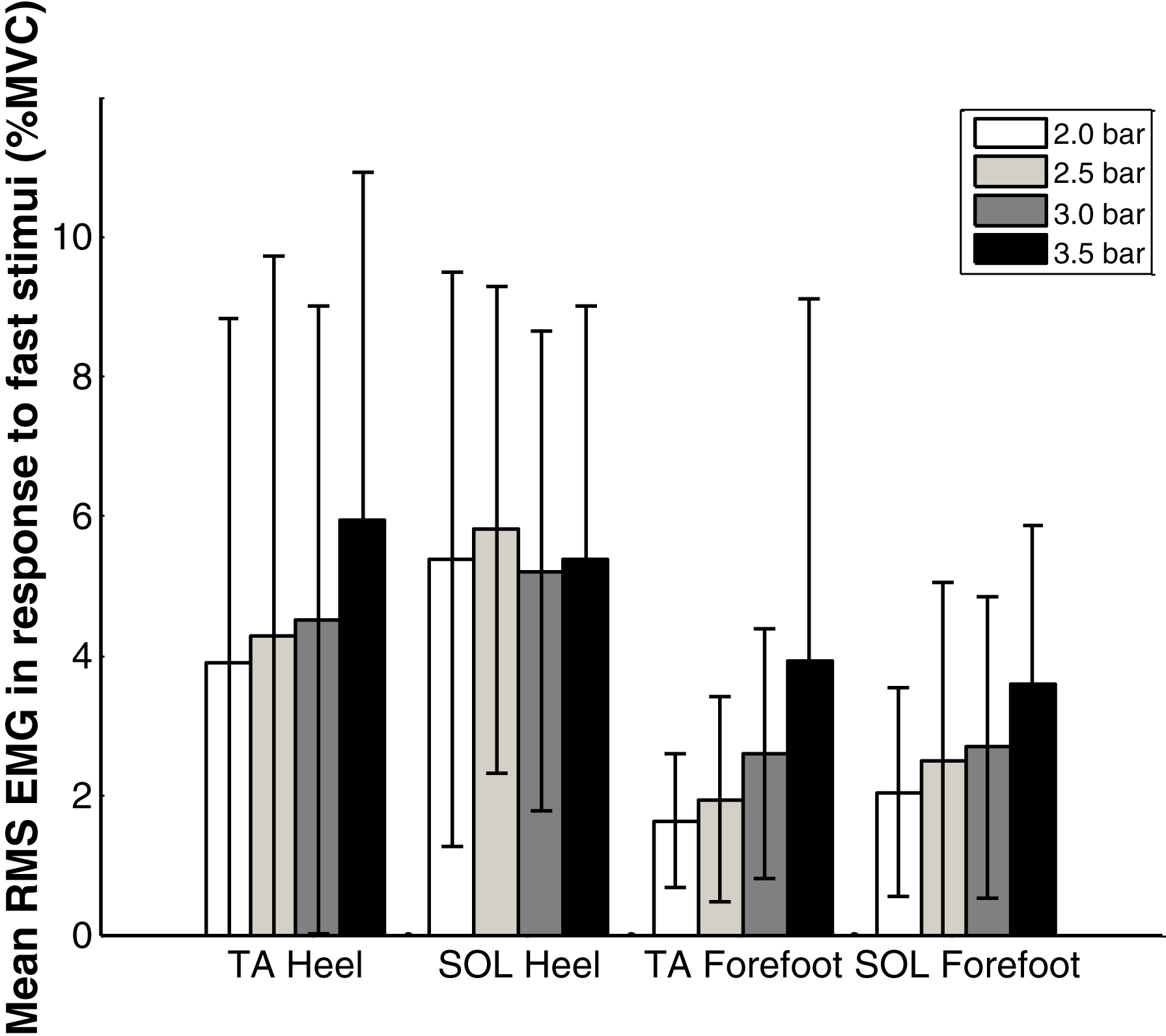

Supplement: Supplementary file 9 — Authors’ original file for figure 9 [file 12984_2014_688_MOESM9_ESM.tif]

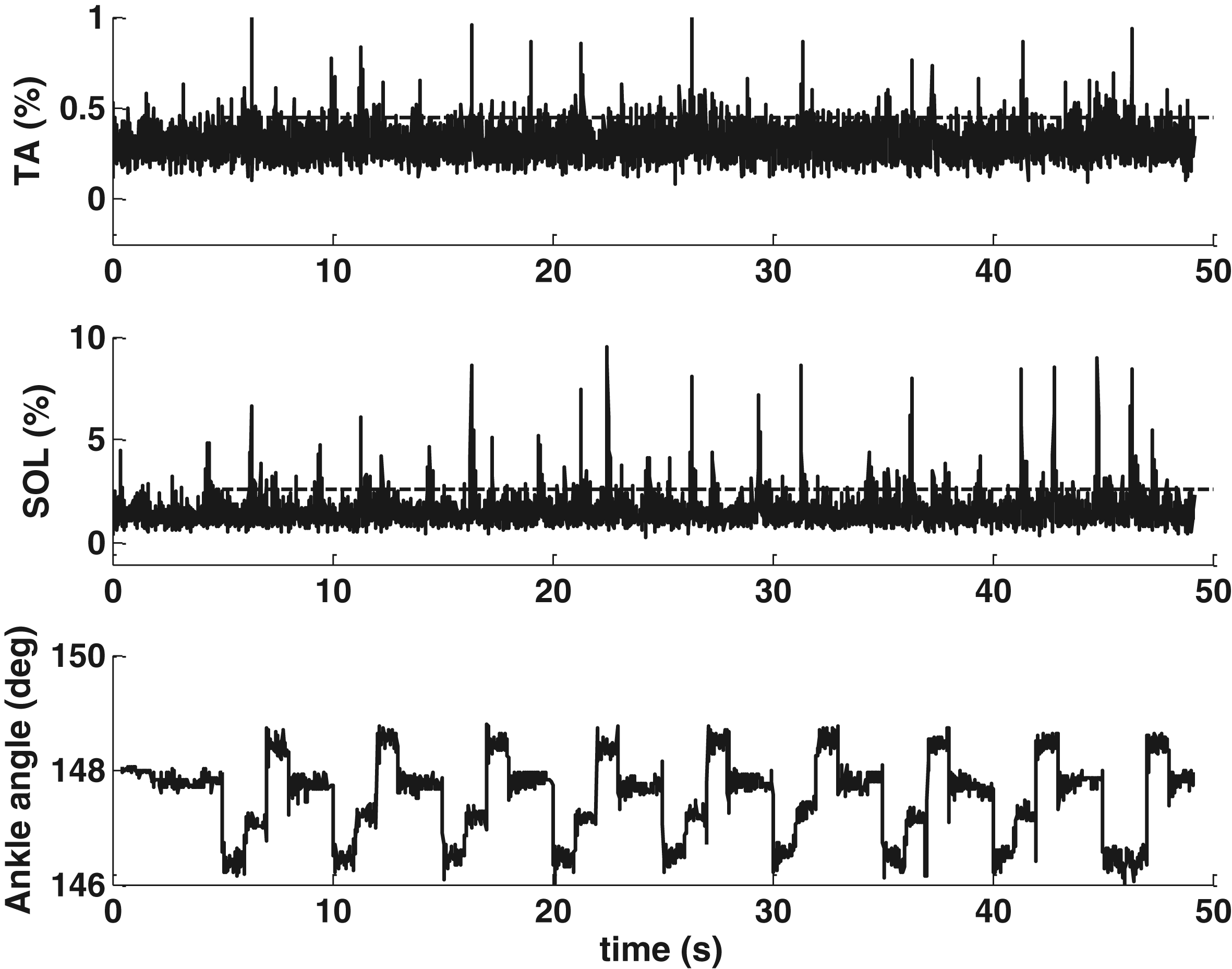

Supplement: Supplementary file 10 — Authors’ original file for figure 10 [file 12984_2014_688_MOESM10_ESM.tif]

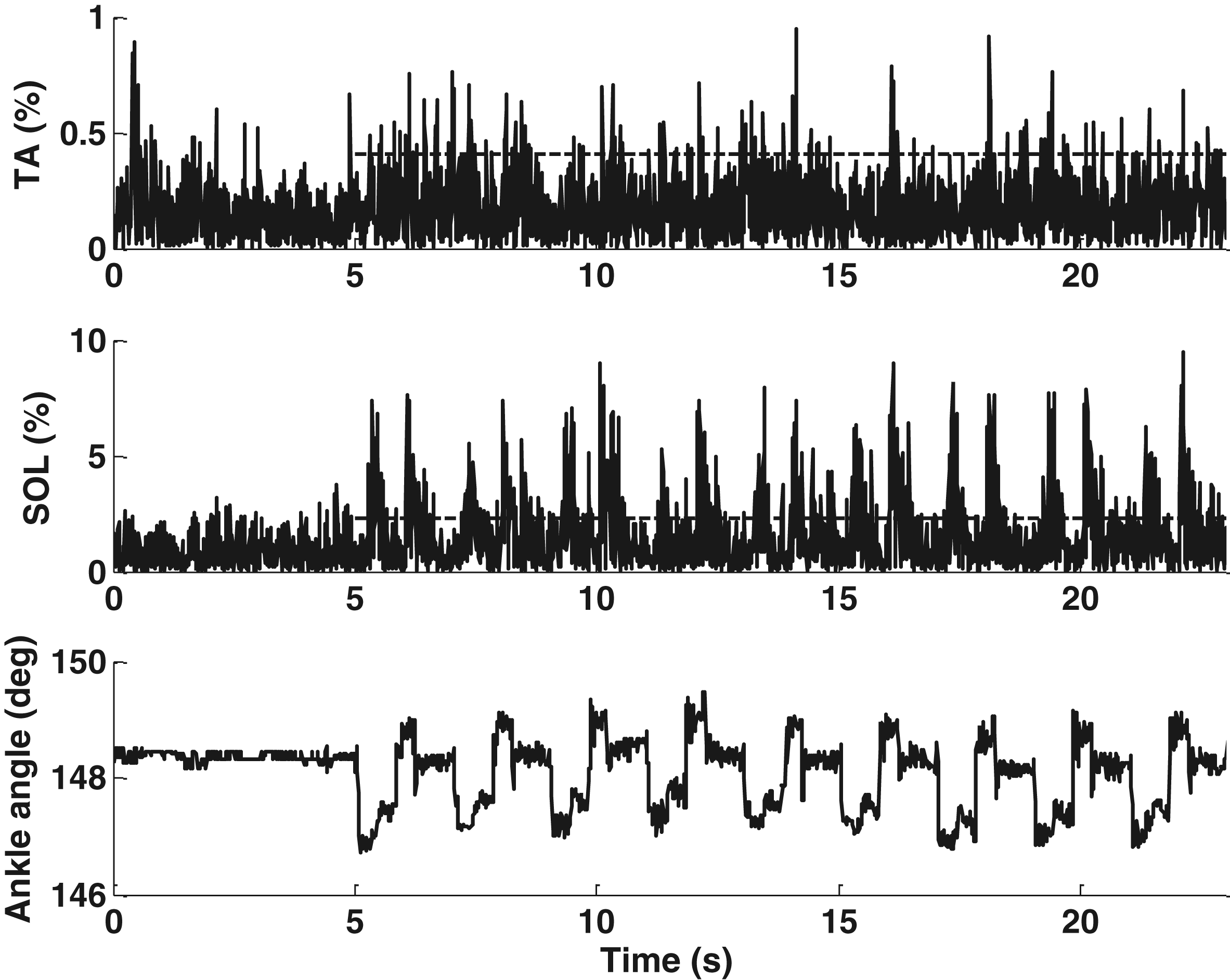

Supplement: Supplementary file 11 — Authors’ original file for figure 11 [file 12984_2014_688_MOESM11_ESM.tif]

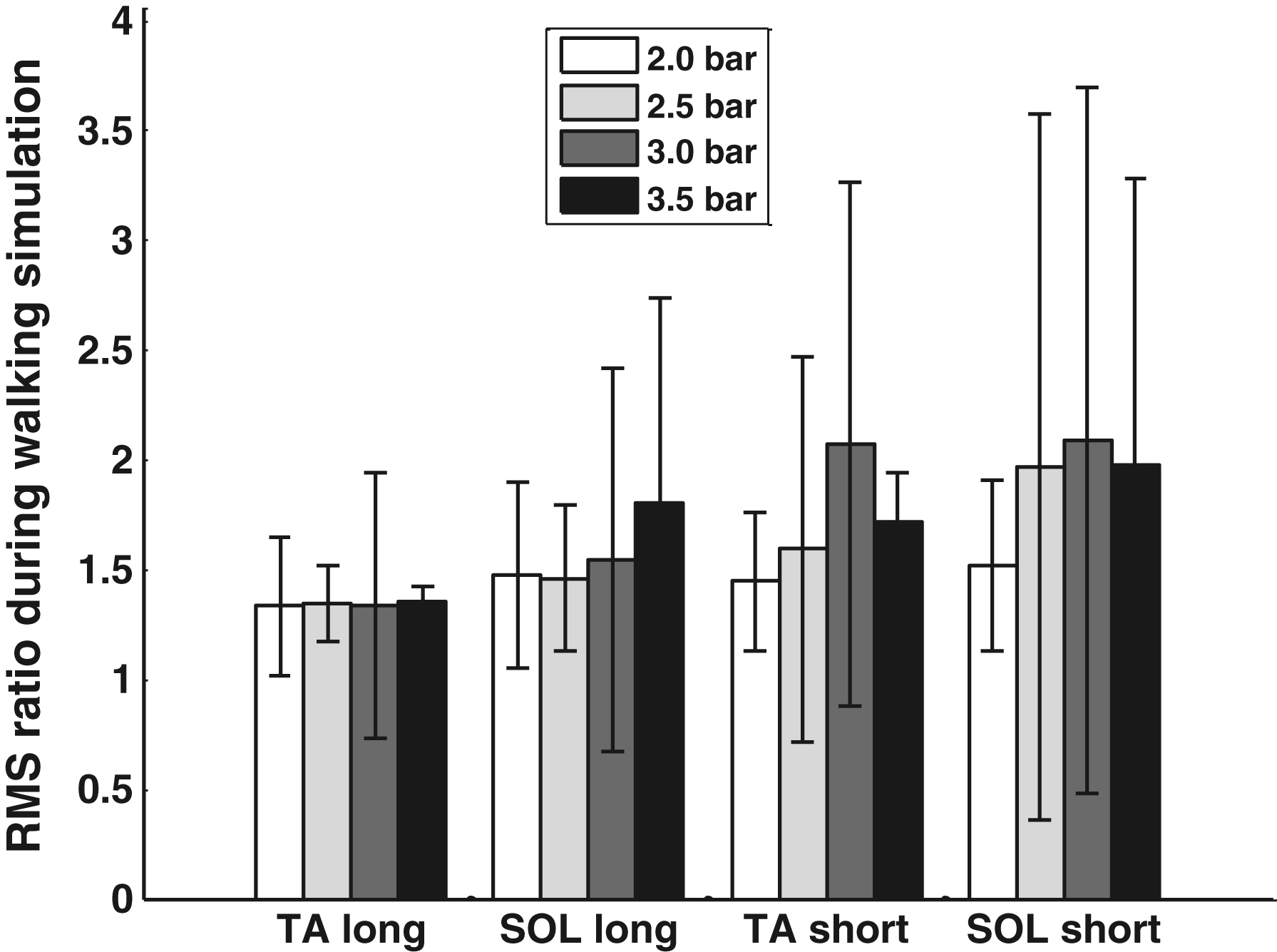

Supplement: Supplementary file 12 — Authors’ original file for figure 12 [file 12984_2014_688_MOESM12_ESM.tif]
